# Host genetics shapes the recovery of the gut microbiome after antibiotic treatment: the role of the blood group related *B4galnt2* gene

**DOI:** 10.1128/msystems.01640-25

**Published:** 2026-04-30

**Authors:** Aleksa Čepić, Philipp Rausch, Theresa Geese, Astrid Dempfle, Guntram A. Grassl, John F. Baines

**Affiliations:** 1 Max Planck Institute for Evolutionary Biology 28319, Plön, Germany; 2 Section of Evolutionary Medicine, Institute for Experimental Medicine, Kiel University 266837, Kiel, Germany; 3 Institute of Clinical Molecular Biology, Kiel University 9179, Kiel, Germany; 4 Institute of Medical Informatics and Statistics, Kiel University and University Hospital Schleswig-Holstein, Kiel, Germany; 5 Institute of Medical Microbiology and Hospital Epidemiology, Hannover Medical School and German Center for Infection Research (DZIF), Hannover, Germany; National Cancer Institute Center for Cancer Research, Bethesda, Maryland, USA

**Keywords:** gut microbiota, antibiotics, streptomycin, *B4galnt2*, host genetics, glycosylation, mouse

## Abstract

The intestinal microbiota is integral to host health, metabolism, and colonization resistance. Antibiotics can disrupt microbial homeostasis, leading to dysbiosis and altered colonization resistance. While antibiotic-induced microbiota disruption is well-documented, less is known about how host genetics shapes post-antibiotic recovery. Here, we investigate the impact of *B4galnt2*, a blood-group-related glycosyltransferase gene, on microbiota recovery following antibiotic treatment. Using a longitudinal, multi-omic approach—including 16S rRNA gene sequencing, metagenomics, and metatranscriptomics—we compare the microbiota dynamics of *B4galnt2^+/–^* and *B4galnt2^–/–^* mice after treatment with streptomycin, kanamycin, and vancomycin. Our findings reveal that *B4galnt2^–/–^* mice exhibit faster recovery of microbial diversity and composition following streptomycin treatment compared to their *B4galnt2^+/–^* counterparts. This accelerated recovery is associated with higher relative abundance of taxa such as *Blautia*, *Dorea,* and other Lachnospiraceae, and increased expression of motility-related genes, and differential regulation of antibiotic resistance genes (ARGs), including the aminoglycoside nucleotidyltransferase genes *aadA* and *aadE*. Genotype-dependent differences in recovery were most pronounced following streptomycin and were not consistently observed with kanamycin or vancomycin, indicating an antibiotic-by-genotype interaction shaped by the *B4galnt2*-associated microbiota. These results underscore the role of host genetics in shaping microbiota response and recovery following antibiotic exposure. By demonstrating the interplay between glycosylation-mediated microbiota composition, antibiotic response, and microbial recovery, our study may provide insights into the potential for personalized approaches to mitigate dysbiosis-related health outcomes.

**IMPORTANCE:**

Antibiotic treatments disrupt the gut microbiome, often leading to long-term alterations that potentially affect host health. While much is known about how antibiotics cause microbial dysbiosis, little is understood about the factors that could influence the speed of microbial community recovery, such as host genetic differences. Using a mouse model, this study reveals that genetic variation at the blood group-related *B4galnt2* gene significantly alters recovery after streptomycin treatment. Mice lacking intestinal *B4galnt2* expression recover faster, with distinct changes in microbial composition, activity, and antibiotic resistance gene expression. These findings highlight how a single host gene can shape microbiota dynamics following antibiotic-induced disruption. The work emphasizes the importance of considering host genetic factors when predicting microbiome responses to antibiotics and suggests potential for genotype-guided strategies to reduce the adverse effects of microbiome-targeted therapies.

## INTRODUCTION

The gastrointestinal tract carries a highly diverse and complex community of microorganisms known as intestinal microbiota. This community plays an important role in nutrient metabolism, immune system development, and protection against invading pathogens (1–4). The composition and diversity of intestinal microbiota vary across regions of the gut and are influenced by factors such as diet, environmental conditions, and host genetic background (5–7).

While critical for controlling bacterial infections, antibiotics are a major disruptor of the intestinal microbiota (8). Their administration induces dysbiosis, characterized by changes in microbiota composition, loss of keystone taxa, and reduced colonization resistance (9–11). These changes have been associated with increased susceptibility to intestinal infections by opportunistic and pathogenic bacteria, including enteric pathogens such as *Salmonella enterica* serovar Typhimurium (11–15).

Antibiotics such as streptomycin, kanamycin, and vancomycin are commonly used in animal models to disrupt microbial homeostasis and establish susceptibility to enteric pathogens (15–17). A single oral dose of streptomycin effectively reduces colonization resistance, enabling *Salmonella* Typhimurium to colonize the gut and induce colitis (15, 18–26). Similarly, kanamycin, with a mechanism of action similar to streptomycin, and vancomycin, an antibiotic with a distinct mechanism, are also used to disrupt colonization resistance in *Salmonella* infection models (16, 17). The resistance against *Salmonella* infection observed in mice with intact microbiota, along with the complete susceptibility of germ free mice, underscores the protective role of the native microbial communities (21, 27).

Interactions between the intestinal microbiota and invading pathogens are also modulated by host genetics. Blood group-related glycosyltransferase genes such as *B4galnt2* (Beta-1,4-N-Acetyl-Galactosaminyltransferase 2) shape glycan structures within the gastrointestinal mucosa and greatly influence microbial composition and host susceptibility to pathogens (19, 28, 29). Notably, variation in tissue-specific expression patterns of *B4galnt2* among wild and laboratory mice alters microbiota-mediated resistance to enteric pathogens, with the loss of gut-specific *B4galnt2* expression (*B4galnt2^–/–^*) associated with reduced susceptibility to intestinal infection (19, 29). While this work revealed *B4galnt2* glycosylation pattern to impact infection outcomes through changes in the intestinal microbiota, the effect is dependent on oral streptomycin treatment before infection (19). Interestingly, the findings suggest that the observed effect may not be solely dependent on tissue specific *B4galnt2* expression. Rather, the expression of *B4galnt2* itself shapes the composition of the commensal microbiota, which in interaction with streptomycin influences the outcome of the *Salmonella* infection (19). However, the mechanisms underlying the microbiota-dependent antibiotic response phenotype driven by intestinal *B4galnt2* expression remain unclear.

In this study, we investigate the impact of *B4galnt2* expression on the composition and function of the commensal microbiota over the course of three different antibiotic treatments using a longitudinal, multi-omic approach, including 16S rRNA gene amplicon-, shotgun metagenomic-, and metatranscriptomic sequencing. As our previous observation of *B4galnt2* genotype-dependent interactions between microbiota, antibiotic treatment, and *Salmonella* infection was made with streptomycin, we here include (i) streptomycin, (ii) kanamycin as a second aminoglycoside, and (iii) vancomycin as a glycopeptide antibiotic with a different mechanism of action, which together enable the generality of the observed effects to be investigated. We observe numerous underlying differences in both the presence and expression of antibiotic resistance genes in *B4galnt2* knockout mice, which associate with differences in recovery time after antibiotic treatment. These results underscore the significance of host genetic factors, which can dictate the pace of post-antibiotic recovery and may inform genotype-guided antibiotic selection.

## RESULTS

**Fig 1:**
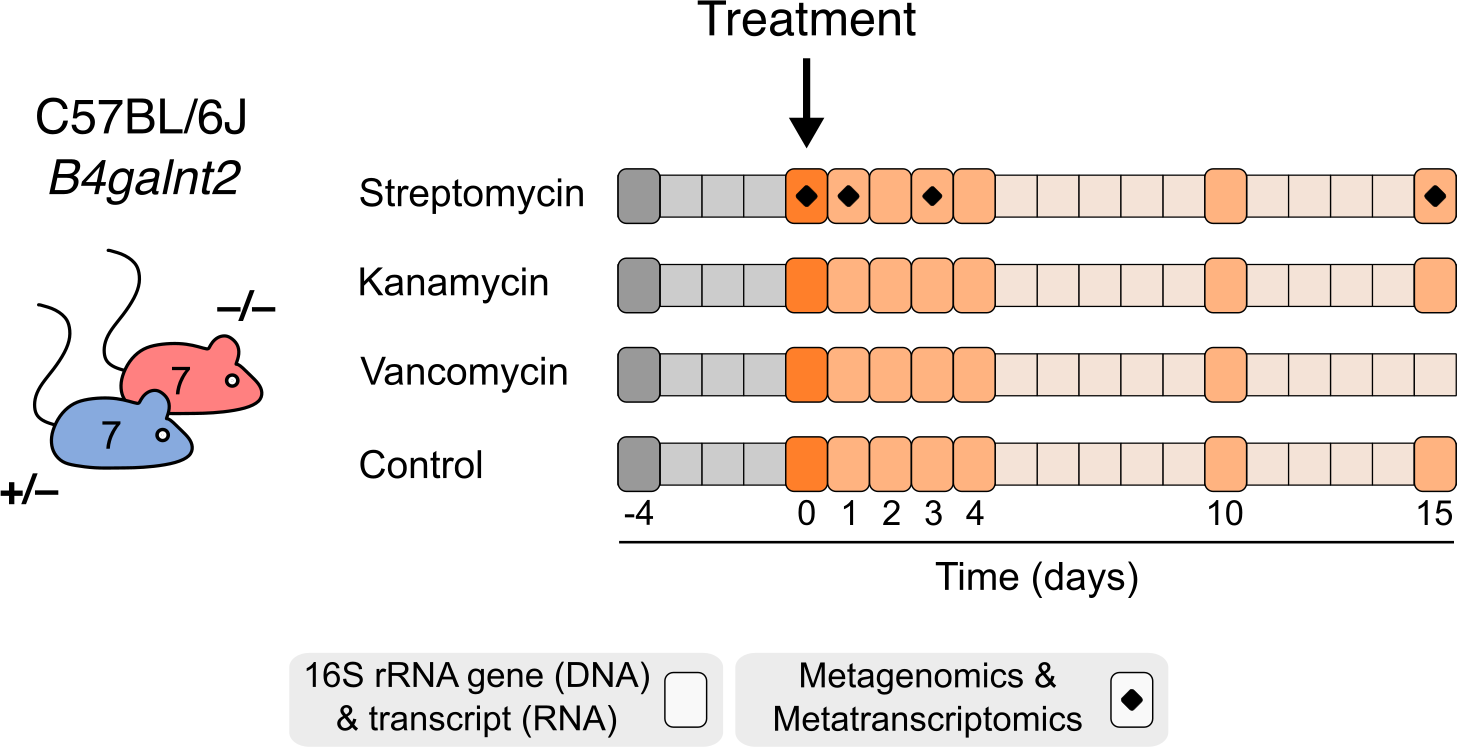
Outline of animal experiment. Each treatment group consisted of *n* = 7 *B4galnt2^+/–^* and *n* = 7 *B4galnt2^–/–^* mice.

To evaluate the longitudinal dynamics of recovery from antibiotic treatment according to *B4galnt2* genotype, *n* = 28 *B4galnt2*^+/–^ and *n* = 28 *B4galnt2*^–/–^ were randomly assigned to one of four treatment groups: control (Ctrl), streptomycin (STR), kanamycin (KAN), and vancomycin (VAN) groups. The antibiotics were administered in a single oral gavage with streptomycin (20 mg) (15, 19, 21, 23), kanamycin (10 mg) (16), and vancomycin (40 mg) (17, 30) per mouse. Fecal samples were collected before, during, and after the antibiotic treatment on days −4, 0, 1, 2, 3, 4, 10, and 15 (Fig. 1) and subject to multiomic analyses.

### 16S rRNA gene (DNA) and transcript (RNA) sequencing reveals genotype-specific community recovery dynamics following streptomycin treatment

Relative microbial abundances were determined by 16S rRNA gene (DNA) sequencing, while active community members were determined by 16S rRNA transcript (RNA) sequencing (31). To document consistency between 16S rRNA libraries, ZymoBIOMICS Microbial Community DNA Standard was used in each sequencing library (Fig. S1). Rarefaction analysis confirmed sufficient sequencing depth at 4,500 reads per sample (Fig. S2).

**Fig 2:**
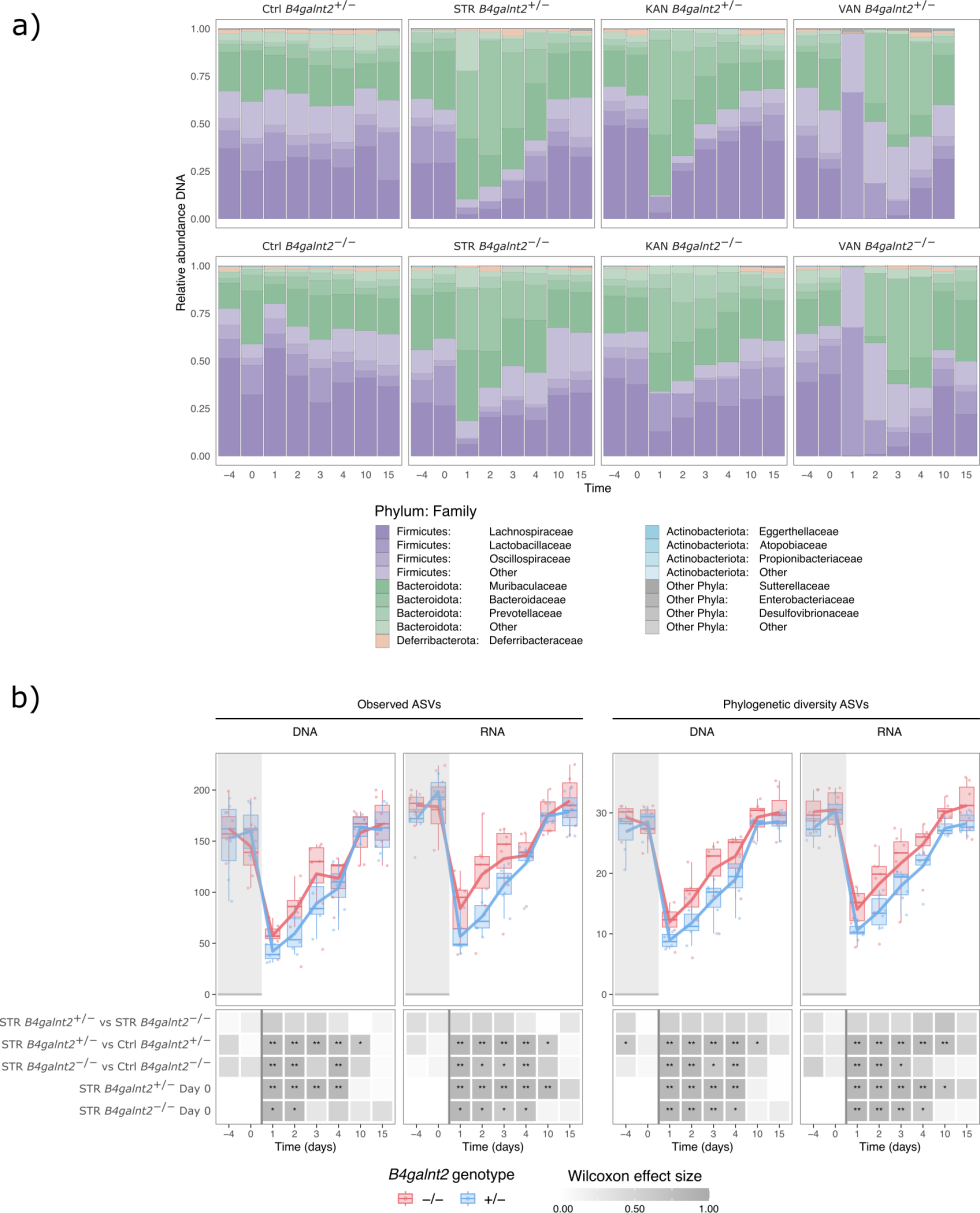
(**a**) Fecal microbiome composition dynamics based on 16S rRNA gene (DNA) sequencing: relative sequence abundance in *B4galnt2*-associated microbial communities at the phylum and family levels; bars represent the mean relative abundance of taxa across all available mice within each genotype and timepoint; (**b**) ASV richness and phylogenetic diversity (PD) in the streptomycin treated mice at the 16S rRNA gene (DNA) and transcript (RNA) levels. Stars denote significance: *P_adj_ < 0.05, **P_adj_  < 0.01, ***P_adj_  < 0.001.

Prior to antibiotic treatment, 16S rRNA gene sequencing analysis revealed dominance of the phyla Bacteroidetes (STR −4: 41.89% ± 16.9%; STR 0: 37.34% ± 13.68%) and Firmicutes (STR −4: 55.71% ± 14.78%; STR 0: 61.75% ± 13.18%) (Fig. 2a). The microbiota’s response to streptomycin treatment exhibited similar trends for both *B4galnt2* groups. Streptomycin administration resulted in a significant decrease in Firmicutes (18.55% ± 8.95%; Maaslin2: *q*_val_ < 0.0001) and a relative increase in Bacteroidetes (80.54% ± 9.36%; Maaslin2: *q*_val_ < 0.0001) by day 1 post treatment (1 dpt), with recovery to baseline levels by 10 dpt in both genotypes.

Similar dynamics were observed in kanamycin-treated mice, with slightly faster recovery (Fig. 2a). In contrast, vancomycin treatment initially reduced Bacteroidetes to 0.39% ± 0.98% (Maaslin2: *q*_val_ < 0.0001), while Firmicutes accounted for the majority of the microbial population by 1 dpt (99.36% ± 0.93%; Maaslin2: *q*_val_ < 0.0001). However, by 10 dpt, the relative abundance of Bacteroidetes returned to pre-antibiotic levels (41.16% ± 13.67%; Maaslin2: *q*_val_ = 0.49).

Fecal microbiota activity, as measured by 16S rRNA transcript sequencing, mirrored these trends observed in the genomic 16S rRNA analyses, but with higher relative proportions of Firmicutes compared to DNA-based sequencing (Fig. S3).

Alpha diversity, as assessed by the number of observed ASVs and the phylogenetic diversity (PD) index, decreased following streptomycin treatment in both *B4galnt2* groups (Fig. 2b). Although no significant differences in ASV richness or PD were observed between *B4galnt2* genotypes at any given time point, within-genotype comparisons to baseline and controls indicated an earlier normalization of the communities in *B4galnt2^−/−^* compared to *B4galnt2^+/−^,* at both the DNA and RNA levels (Fig. 2b). This differential recovery pattern was, however, not consistently observed in the other antibiotic treatments (Fig. S4 and S5).

**Fig 3:**
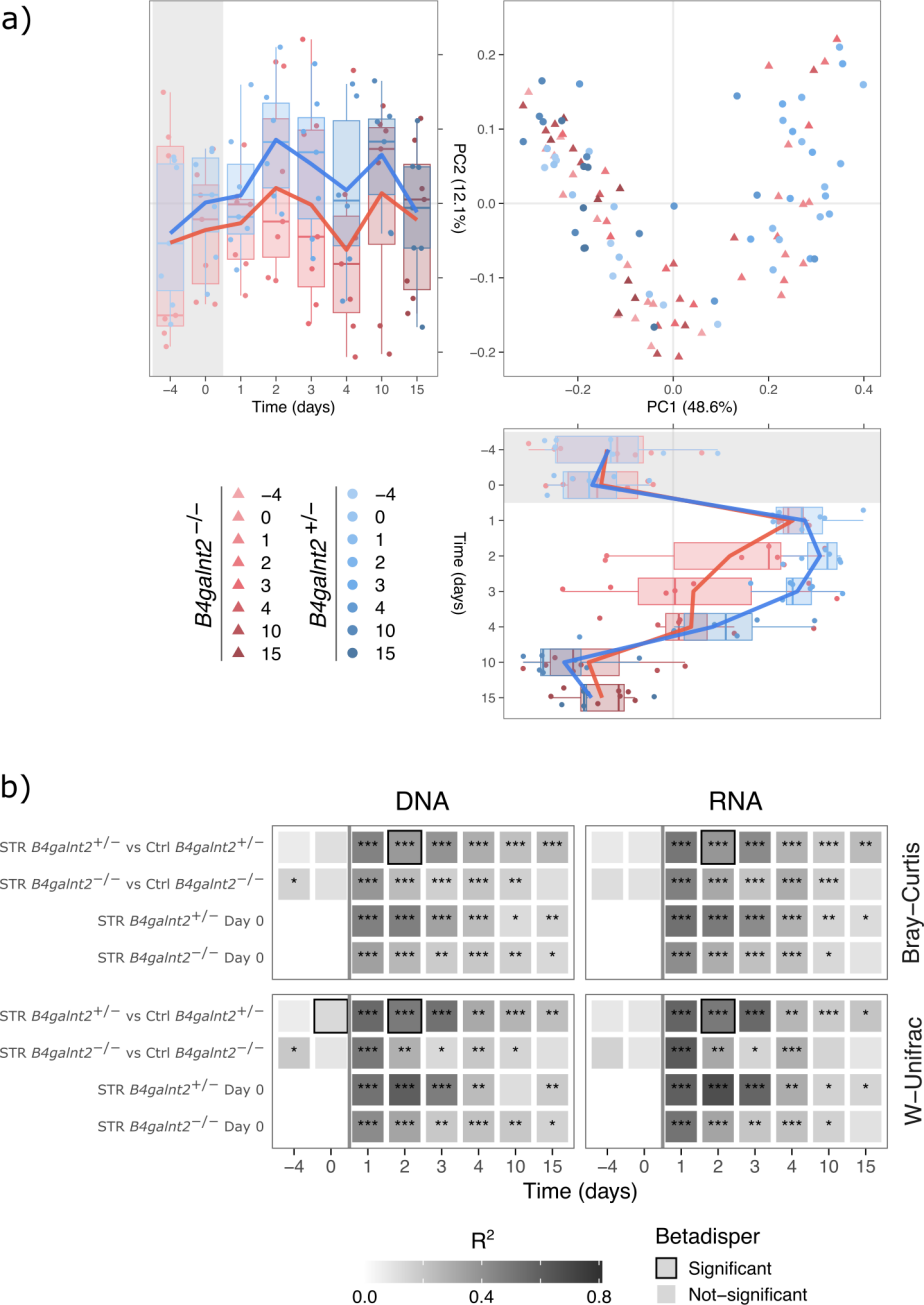
(**a**) Principal coordinate analysis (PCoA) plot of samples before, during, and after the streptomycin treatment based on weighted Unifrac (W-UniFrac) distances at the 16S rRNA transcript (RNA) level. The distribution of samples by *B4galnt2* groups and time is shown along the first and second axes of the PCoA plot. (**b**) Significance and effect size estimates for PERMANOVA and Betadisper analysis for differences in the streptomycin treated mice at 16S rRNA gene (DNA) and transcript (RNA) levels between *B4galnt2* genotypes. Stars denote significance: *P_adj_ < 0.05, **P_adj_  < 0.01, ***P_adj_  < 0.001.

Beta diversity analysis based on Bray-Curtis and weighted Unifrac distance metrics revealed similar dynamics at both the 16S rRNA gene and transcript levels (Fig. 3a; Fig. S6 to S8). Both the *B4galnt2*^+/–^ and *B4galnt2*^–/–^ groups clustered together at baseline. After the administration of streptomycin at 1 dpt, both genotypes exhibited a significant compositional shift compared to baseline. Ordination plots (PCoA; Fig. 3a) suggest that *B4galnt2^–/–^* communities began trending back toward baseline composition as early as 2 dpt, whereas *B4galnt2^+/–^* communities display a less pronounced recovery trajectory. However, PERMANOVA analysis (Fig. 3b) indicated that full recovery—defined as no significant difference from baseline or controls—was achieved by 15 dpt in *B4galnt2^–/–^* mice, while *B4galnt2^+/–^* mice still differed significantly from baseline at this time point. Kanamycin treatment shows broadly similar visual trajectories; however, no consistent genotype-specific recovery is detected when compared to genotype-matched controls or baseline composition at either the 16S rRNA gene or transcript levels (Fig. S9 to S12). Vancomycin induced greater compositional perturbations, but recovery rates remained similar between genotypes across 16S DNA/RNA profiles (Fig. S13 to S16).

To further validate the significance of the observed patterns for streptomycin treatment, we performed PERMANOVA analyses of Bray-Curtis and weighted UniFrac distances, taking into account potential sex effects. Direct comparison between genotypes revealed statistically significant differences only at the 16S rRNA gene level at 10 dpt (Bray–Curtis: PERMANOVA, *R*^2^ = 0.1288; *P*_adj_ = 0.0360; W–Unifrac: PERMANOVA, *R*^2^ = 0.1540; *P*_adj_ = 0.0208). When comparing the *B4galnt2* genotypes with their respective controls, both groups exhibited significant changes after treatment. Notably, the *B4galnt2*^–/–^ recovered faster at both the 16S rRNA gene and transcript levels (Fig. 3b). A similar trend of faster community recovery in *B4galnt2*^–/–^ mice was also observed at the 16S rRNA transcript level.

Mice treated with kanamycin showed a similar recovery trend to the streptomycin group when compared to controls, with faster recovery in *B4galnt2*^–/–^ mice. However, the kanamycin-treated mice did not exhibit differential recovery between genotypes when compared to their respective baseline at either the 16S rRNA gene or transcript level (Fig. S17). The vancomycin-treated mice also displayed no significant differences in recovery time according to *B4galnt2* genotype (Fig. S18).

Finally, network-based analyses were performed for both 16S rRNA gene and transcript-derived data sets to examine community dynamics through co-occurrence patterns. As expected, antibiotic treatments resulted in numerous alterations in network properties (Fig. S19), but we here focus specifically on differences in network properties with respect to *B4galnt2* genotype among the treated mice. In the streptomycin-treated *B4galnt2^–/–^* group, communities were characterized by increased clustering coefficients and edge density during early recovery, together with reduced modularity and reduced vertex connectivity, compared to the streptomycin-treated *B4galnt2^+/–^* group (Fig. S19a). This combination of properties reflects networks with tightly interconnected local communities but weaker global separation, which is an architecture previously associated with increased robustness and faster reassembly following perturbations (32). For kanamycin-treated *B4galnt2*^–/–^ mice, several similar characteristics were detected, including a relative reduction in average path length and increased clustering coefficient, edge density, and natural connectivity during early recovery, compared to the kanamycin-treated *B4galnt2^+/–^* group (Fig. S19b). Furthermore, the network characteristics in *B4galnt2*^–/–^ mice appear to change in a less variable pattern than observed in *B4galnt2^+^*^/–^ mice during recovery. These properties suggest increased overall connectivity and cohesion within the communities during the early recovery phase (more compact network), albeit through a network configuration that differs from that observed under streptomycin, with an on average stronger change of network properties. In contrast, vancomycin-treated mice exhibited only minimal genotype-specific differences (Fig. S19c), and their network properties did not recapitulate the patterns observed under either streptomycin or kanamycin treatment, suggesting that the altered network signatures are specific to the two aminoglycoside antibiotics.

Thus, overall, the results based on 16S rRNA profiles (DNA and RNA based) indicate that genotype-specific recovery dynamics are most clearly detectable under streptomycin treatment, with some patterns also observed for kanamycin, and little to no effect observed for vancomycin.

### Shotgun metagenomics confirms accelerated recovery of diversity and taxonomic shifts in *B4galnt2^–/–^* mice

**Fig 4:**
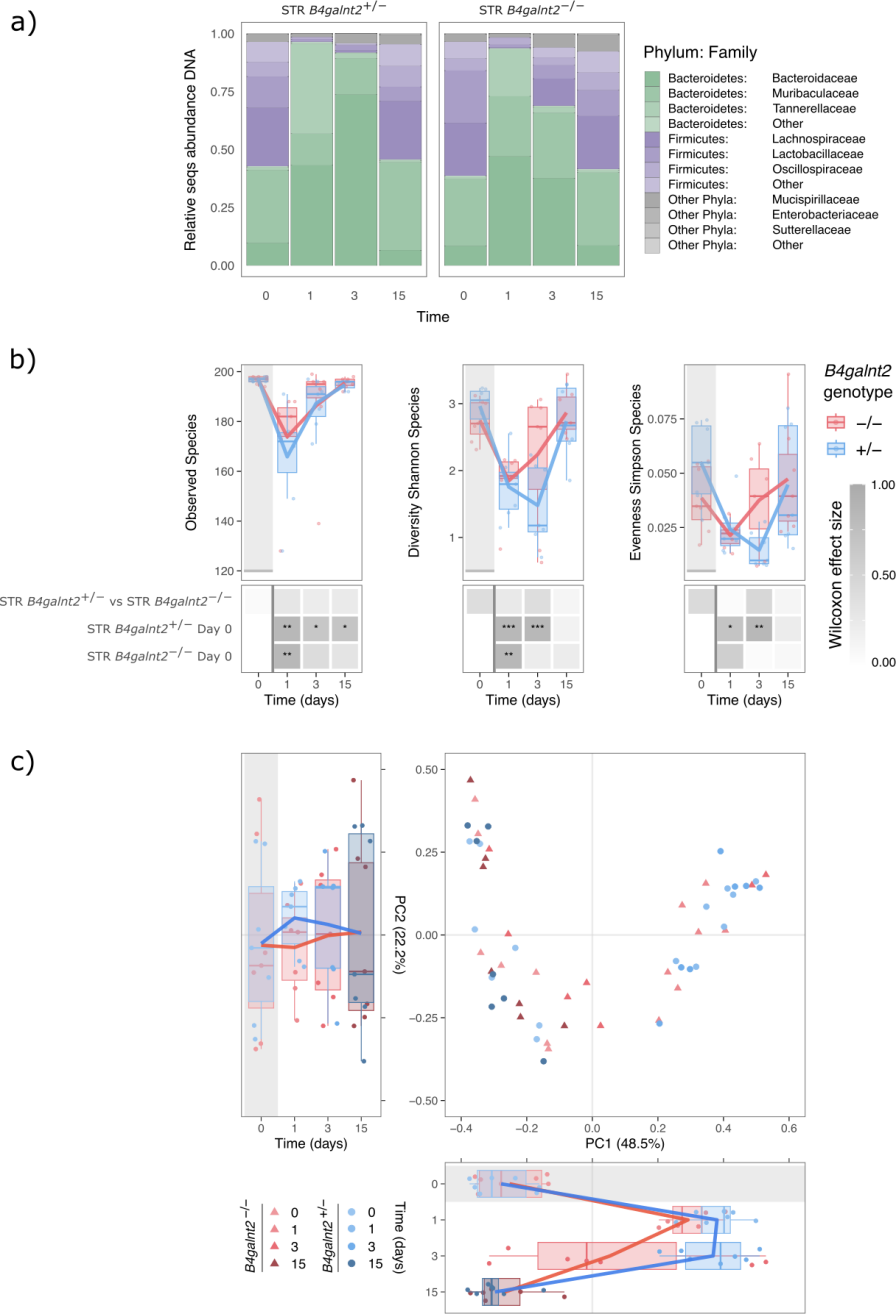
(**a**) Fecal microbiome composition dynamics based on metagenomics (MGX) sequencing: relative sequence abundance in *B4galnt2*-associated microbial communities at the phylum and family levels. (**b**) Species richness, Shannon and Simpson diversity indices in the streptomycin treated mice at the metagenomics (MGX) level. Stars denote significance: *P_adj_ < 0.05, **P_adj_  < 0.01, ***P_adj_  < 0.001. (**c**) Principal coordinate analysis (PCoA) plot of samples before, during, and after the streptomycin treatment (STR) based on Bray-Curtis distances at the metagenomics (MGX) level. The distribution of samples by *B4galnt2* groups and time is shown along the first and second axes of the PCoA plot.

Given the intriguing genotype-specific differences in recovery unique to streptomycin, we chose selected samples from the streptomycin treatment group for further in-depth analysis using metagenomics and metatranscriptomics. These data sets included samples before (0 dpt), early recovery (1 and 3 dpt), and late recovery (15 dpt) time points. At the metagenomic level, the phylum-level composition was consistent with the 16S rRNA gene profiles prior to treatment. Bacteroidetes (38.71% ± 28.34%) and Firmicutes (57.79% ± 26.39%) phyla accounted for most of the relative sequence abundances (Fig. 4a). After the antibiotic treatment, we observed a decrease in the relative abundance of Firmicutes (4.72% ± 4.39%), which showed relative recovery at 3 dpt (25.16% ± 19.94%). By 15 dpt, the relative abundances of Bacteroidetes (41.58% ± 24.55%) and Firmicutes (50.73% ± 20.19%) returned to pre-antibiotic levels for both *B4galnt2* genotypes.

Next, we evaluated alpha diversity according to *B4galnt2* genotype before, during, and after the streptomycin treatment using richness, Shannon, and Simpson diversity indices. The administration of streptomycin on 0 dpt resulted in a significant and immediate reduction in microbial species richness and diversity by 1 dpt. On 3 dpt, *B4galnt2*^–/–^ mice showed a recovery of microbiota diversity to pre-antibiotic levels, while *B4galnt2*^+/–^ mice achieved full recovery at 15 dpt (Fig. 4b).

Linear mixed-effects models applied to the metagenomic data set did not detect a genotype × time interaction for observed richness (*P*_val_ = 0.595) but identified significant interactions for Shannon diversity (*P*_val_ = 0.045) and Simpson evenness (*P*_val_ = 0.005). Between-genotype analyses showed higher Shannon diversity (*P*_adj_ = 0.029) and evenness (*P*_adj_ = 0.046) in *B4galnt2^−/−^* mice than *B4galnt2^+/−^* at 3 dpt, while no differences were observed at baseline, 1 dpt, or 15 dpt (Table S1). Within-genotype analyses revealed that Shannon diversity decreased on 1 dpt in both groups, but only *B4galnt2^−/−^* mice returned to baseline levels by 3 dpt (*P*_adj_ = 0.101), whereas *B4galnt2^+/−^* mice remained at reduced diversity levels (*P*_adj_ = 2.6 × 10⁻⁶). Evenness remained unaltered in *B4galnt2^−/−^* mice (1 dpt: *P*_adj_ = 0.479; 3 dpt: *P*_adj_ = 0.920), but decreased in *B4galnt2^+/−^* at 1 dpt (*P*_adj_ = 0.032) and day 3 (*P*_adj_ = 0.0019). By 15 dpt, both indices returned to baseline, supporting a pattern of continuous and faster community recovery in *B4galnt2^−/−^* mice (Table S2).

Prior to streptomycin treatment, microbial communities of both *B4galnt2* genotypes cluster together in a beta diversity analysis (Fig. 4c; Table 1). One day after the antibiotic treatment, the microbial communities underwent a significant shift in composition, and began recovery to their initial state by 3 dpt. The *B4galnt2*^–/–^ group exhibited faster recovery when compared to their baseline level (STR^+/–^ vs 0 dpt: PERMANOVA, *R*^2^ = 0.6204; *P*_adj_ = 0.0005; STR^–/–^ vs 0 dpt: PERMANOVA, *R*^2^ = 0.1124; *P*_adj_ = 0.1124). By 15 dpt, the microbial communities of both *B4galnt2* genotypes returned to clustering with their respective baseline samples.

**TABLE 1:** PERMANOVA and Betadisper results of species relative abundances based on Bray–Curtis distances for the streptomycin treated mice at metagenomics (MGX) level^*a*^

Time	STR *B4galnt2^+/–^* vs day 0			STR *B4galnt2^–/–^* vs day 0		
	*P*_adj_	*R* ^2^	Dispersion	*P*_adj_	*R* ^2^	Dispersion
1	**0.0005**	0.6536	0.3451	**0.0006**	0.3979	0.5607
3	**0.0005**	0.6204	0.5320	0.1124	0.1642	0.5550
15	0.4200	0.0523	0.6348	0.8230	0.0275	0.8058

^*a*^ Bold values indicate statistical significance.

**Fig 5:**
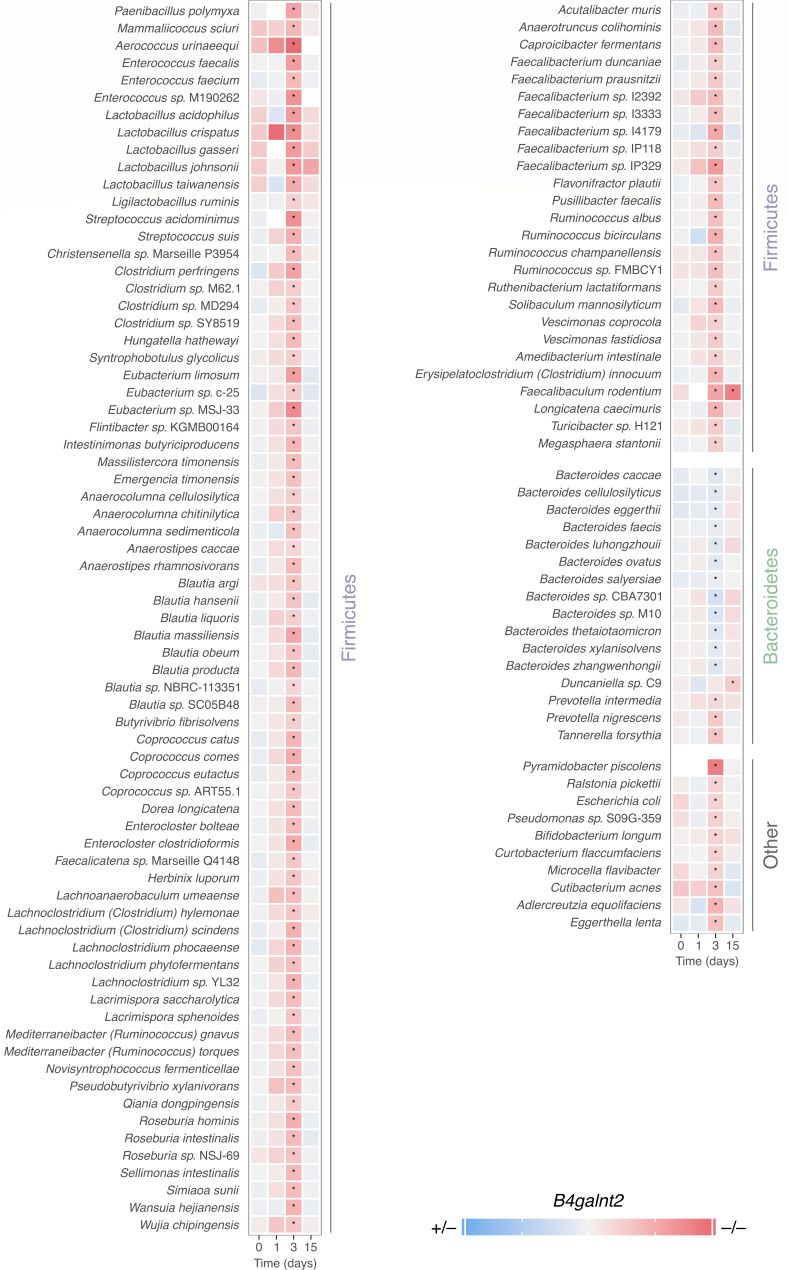
Differently abundant species between *B4galnt2* groups at different time points. Stars represent a *q*_val_ < 0.25.

To identify specific taxonomic signatures associated with *B4galnt2* genotype-dependent differences in streptomycin treatment outcome, we performed multivariate analyses using Maaslin2 (33). A total of 123 species and 73 genera were associated with *B4galnt2* genotype throughout the experiment (Fig. 5; Fig. S20). Most differentially abundant species were specific to the early (3 dpt) recovery stage and enriched in *B4galnt2*^–/–^ mice. Most of these species belonged to the Firmicutes phylum, including the Lachnospiraceae and Oscillospiraceae families. We observed several taxa previously associated with colonization resistance or anti-inflammatory effects including members of the *Blautia* genus (34, 35), *Enterococcus faecalis* (35, 36), *Enterocloster clostridioformis* (35, 37), *Coprococcus eutactus* (38), *Roseburia intestinalis* (39), *Roseburia hominis* (40), *Bifidobacterium longum* (41) among others. In contrast, 12 *Bacteroides* species were specific to early recovery in *B4galnt2*^+/–^ mice (Fig. 5).

### Metatranscriptomics reveals early functional activation and enrichment of flagella in *B4galnt2^–/–^* mice

Functional profiling of shotgun metagenomic and metatranscriptomic sequences was performed using Humann3 (42) in streptomycin treated and untreated mice. Functional community differences based on Bray-Curtis distances of the KEGG Orthology groups (metagenomic level) show that *B4galnt2*^–/–^ mice recover faster after the streptomycin treatment than *B4galnt2*^+/–^ (Table 2). Similarly, at the metatranscriptome level *B4galnt2*^–/–^ mice recovered by 3 dpt, while *B4galnt2*^+/–^ mice continue to display significant differences compared to baseline even after 15 dpt.

**TABLE 2:** PERMANOVA and Betadisper results of the KEGG Ortholog groups based on Bray–Curtis distances for the streptomycin treated mice at metagenomics (MGX) and metatranscriptomic (MTX) levels^*a*^

Time	STR *B4galnt2^+/–^* vs day 0			STR *B4galnt2^–/–^* vs day 0			Level
	*P*_adj_	*R* ^2^	Dispersion	*P*_adj_	*R* ^2^	Dispersion	
1	**0.0006**	0.7151	**0.0286**	**0.0018**	0.6322	0.2144	MGX
3	**0.0006**	0.6197	**0.0371**	0.3512	0.1564	0.2417	
15	0.6652	0.0255	0.9060	0.7938	0.0207	0.9480	
1	**0.0005**	0.6169	**0.0001**	**0.0027**	0.4459	0.0815	MTX
3	**0.0005**	0.6364	0.1694	0.1947	0.1383	0.2043	
15	**0.0011**	0.4219	0.8399	0.2360	0.1077	0.1627	

^*a*^ Bold values indicate statistical significance.

Next, we performed differential abundance analyses using Maaslin2 (33), which revealed significant differences between *B4galnt2* mice in 126 out of 2,774 KEGG Orthology groups, in at least one of the time points. Across all time points, metatranscriptomic (MTX) profiling revealed genotype-associated differences spanning a broad range of microbial functions, including carbohydrate and energy metabolism, amino acid and nucleotide biosynthesis, membrane transport, and stress- and DNA-repair pathways. These functional differences were most pronounced at 3 dpt, when *B4galnt2*^−/−^-associated communities exhibited higher transcript abundances across many pathways, indicating an early genotype-dependent activation of microbial functions during recovery (Table S3). By 15 dpt, these differences were largely attenuated, consistent with a transient transcriptional divergence between genotypes during the critical early recovery phase.

**Fig 6:**
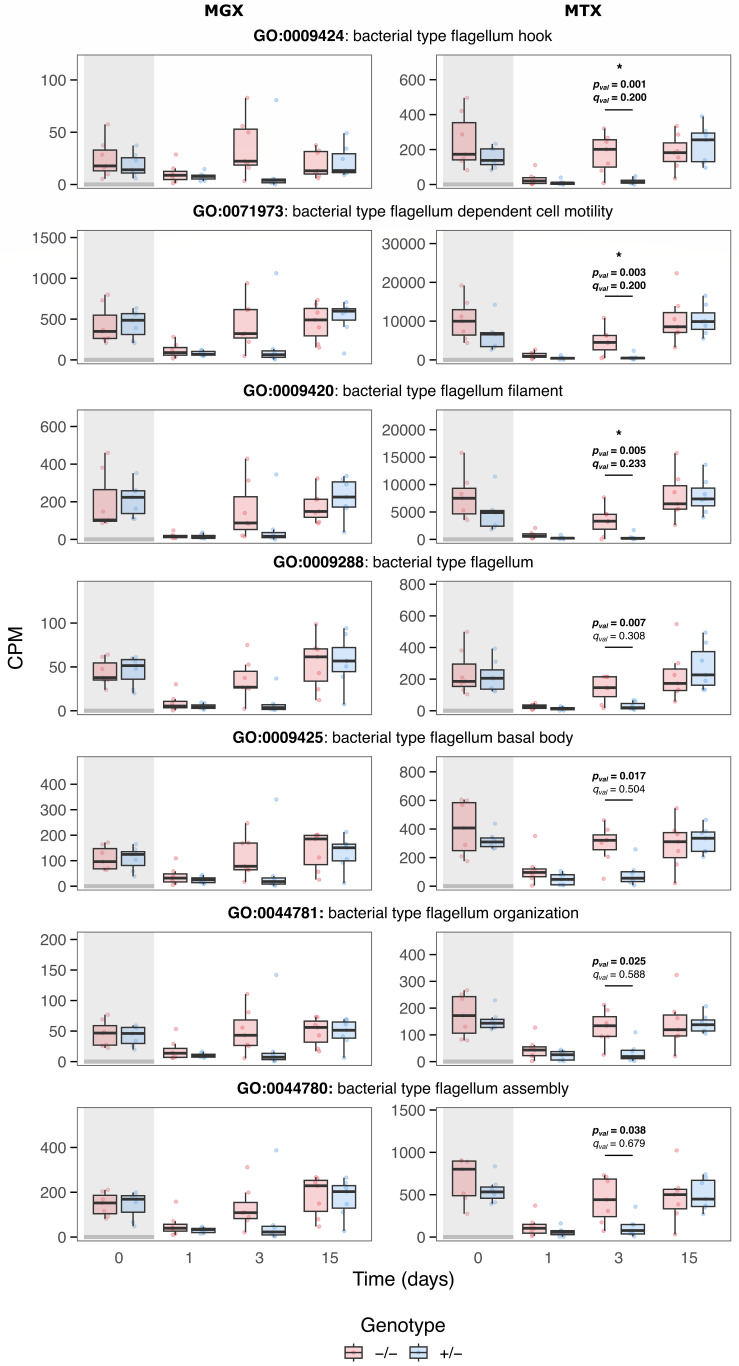
Differential Gene Ontology (GO) terms associated with flagellum organization and motility-related biological processes between *B4galnt2*^+/–^ and *B4galnt2*^–/–^ mice in metagenomics (MGX) and metatranscriptomics (MTX) samples. Stars represent a *q*_val_ < 0.25.

Within this broader functional landscape, Gene Ontology (GO) analysis identified motility- and flagellum-related genes formed one of the most consistent and temporally enriched modules. Among all tested GO terms, seven flagellum-related terms were identified: all seven were nominally significant (*P*_val_ < 0.05), and three out of seven remained significant after multiple-testing correction (*q*_val_ < 0.25) (Fig. 6). These genes showed consistently higher transcript abundance in *B4galnt2*^−/−^ communities specifically at 3 dpt, with no systematic differences at other time points. The temporal pattern of this module paralleled the accelerated recovery phenotype observed in *B4galnt2*^−/−^ mice, suggesting that early activation of motility-associated functions may contribute to, or reflect, more rapid community reassembly after streptomycin treatment (Fig. 6).

### Metatranscriptomics reveals rapid aminoglycoside resistance response in *B4galnt2^–/–^* mice

To perform a targeted analysis of antibiotic resistance genes (ARGs), we mapped metagenomic- and metatranscriptomic reads against a curated ARG database and compared profiles using Maaslin2 (33). This analysis identified notable differences according to *B4galnt2* genotype, both before and after streptomycin treatment.

**Fig 7:**
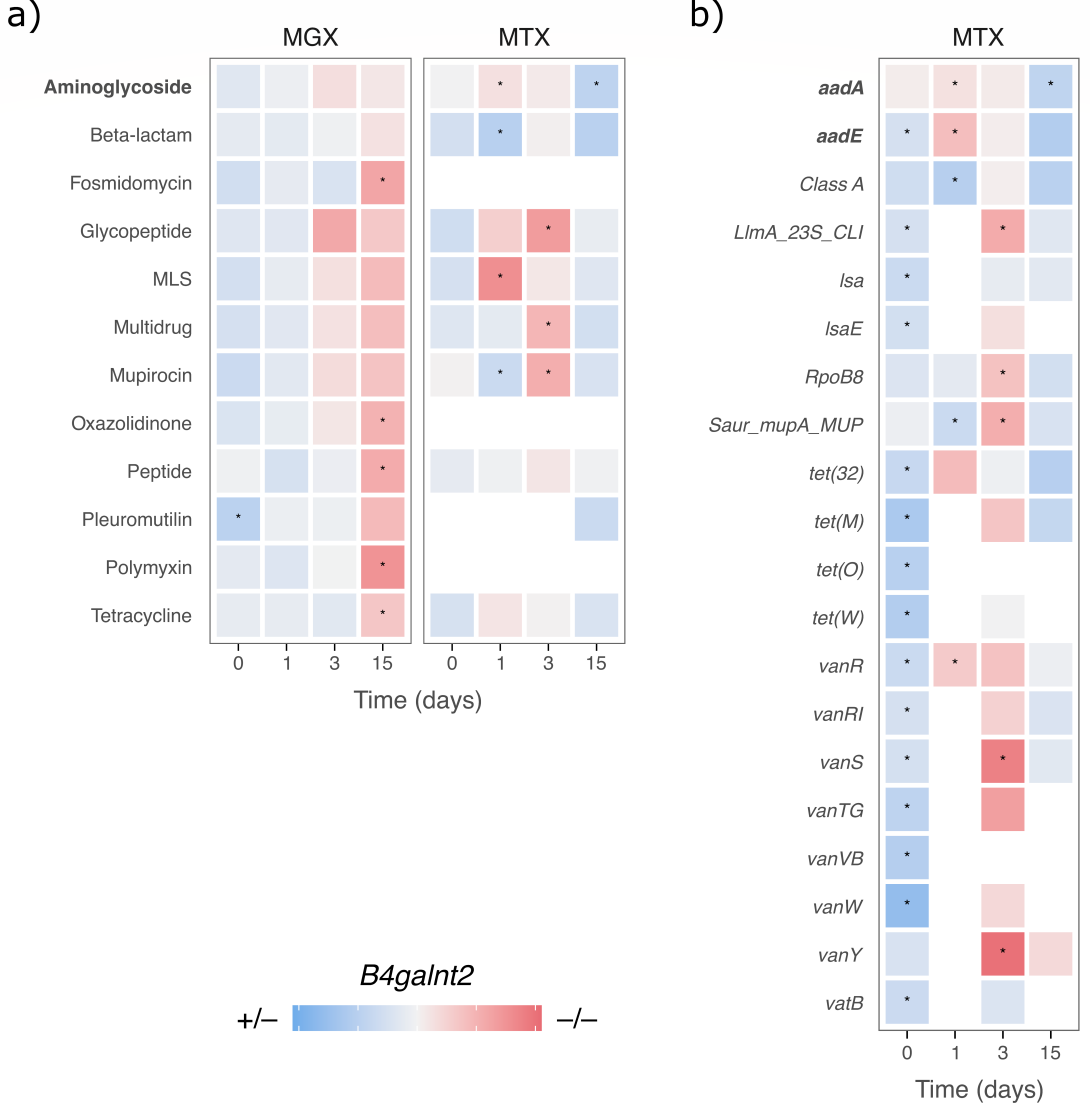
Differential abundance of antibiotic resistance gene classes on MGX and MTX levels (**a**), and differential abundance of antibiotic resistance genes on MTX level (**b**). Stars represent a *q*_val_ < 0.25.

Prior to streptomycin treatment, a higher abundance of pleuromutilin class resistance genes was observed in metagenomic samples of *B4galnt2*^+/–^ mice (Fig. 7a). In contrast, after streptomycin treatment, a notable increase of genes associated with fosmidomycin, oxazolidinone, peptide, polymyxin, and tetracycline classes of antibiotics was observed in *B4galnt2*^–/–^ mice. However, no genotype-specific differences were observed for streptomycin-specific aminoglycoside class resistance genes at the metagenomic level.

In contrast to the metagenomic profiles, metatranscriptomic analyses revealed a higher abundance of expressed aminoglycoside, glycopeptide, MLS, muciporin, and multidrug class related resistance genes in the *B4galnt2*^–/–^ mice during early recovery 1 and 3 dpt. Conversely, beta-lactam and muciporin resistance genes are enriched in *B4galnt2*^+/–^ mice during early recovery (1 dpt), whereas aminoglycoside resistance genes are observed during the later recovery phase (15 dpt, Fig. 7a). Upon analyzing specific antibiotic resistance genes at the metatranscriptome level, we found a higher expression of aminoglycoside nucleotidyltransferase genes (*aadA* and *aadE*) in *B4galnt2*^–/–^ mice 1 dpt. Notably, at 15 dpt, we observed significantly higher transcript levels of *aadA* in *B4galnt2*^+/–^ mice (Fig. 7b). Thus, these findings suggest that the microbial communities of *B4galnt2*^–/–^ mice were able to mount a more rapid transcriptional response to streptomycin treatment compared to *B4galnt2*^+/–^ associated communities, in part through the expression of aminoglycoside nucleotidyltransferase—as well as flagellin-related genes.

### CAZyme analysis reveals *B4galnt2*-associated carbohydrate degradation enzyme dynamics

Given *B4galnt2’s* function as a glycosyltransferase, we next performed an analysis of carbohydrate-active enzymes (CAZyme) in the microbiome. CAZyme profiles displayed significant compositional differences according to *B4galnt2* genotype at different time points after streptomycin treatment, including the beginning and the end of the time course (Fig. S21). This pattern was present in both metagenome- and metatranscriptome-based analyses. In terms of individual CAZymes, their abundance changed over time in a *B4galnt2* genotype specific manner on the genomic- and transcriptomic level (Fig. S22 and S23; Table S4). The abundances of GH13_16, GH13_23, GH30_1, and GT56 increased in *B4galnt2*^+/−^ mice, while the abundance of GT41 and GH165 increased in *B4galnt2*^−/−^ mice. In contrast, the expression of CBM79, CBM12, and GH112 increased in *B4galnt2*^+/−^ mice over time, while GT2, GT20, GT25, GT53, and GH154 increased in *B4galnt2^−/−^* mice. Only GH32 decreased irrespective of genotype, but decreased strongest in *B4galnt2*^+/−^ mice. Overall, these CAZyme families included glycosyltransferases and glycoside hydrolases involved in mucin and complex carbohydrate metabolism, suggesting differential glycan-degrading strategies shaped by *B4galnt2* genotype.

Individual CAZymes also displayed significant differences between genotypes at single time points before and after Streptomycin treatment (DNA: GT56 [0 dpt], GH30_1 [3 dpt], RNA: GT2 [15 dpt]). However, differential abundance analyses did not reach significance after correction for multiple testing (Table S5).

## DISCUSSION

This study investigates the role of intestinal *B4galnt2* expression in shaping the recovery dynamics of gut microbiota following antibiotic treatment, and provides new insights into the interplay between host genetic factors and trajectory of post-antibiotic microbiota recovery. Our findings suggest that the absence of intestinal *B4galnt2* expression accelerates microbial recovery after antibiotic exposure, revealing genotype-specific effects on microbiota composition and functional pathways. This highlights the potential role of host genetics in influencing microbiota recovery dynamics after antibiotic treatment and sheds light on microbiota-mediated mechanisms that may explain the differential susceptibility of *B4galnt2*-deficient mice to intestinal *Salmonella* Typhimurium infection, as observed in previous experiments (19). In addition to disrupting commensal microbiota, streptomycin treatment also induces host-mediated changes, including increased epithelial oxygenation and shifts in nutrient availability, creating a metabolic landscape favorable for *Salmonella* expansion (43, 44). Moreover, dietary factors such as reduced fiber intake and increased fat consumption have been linked to an elevated gut colonization by *Salmonella* Typhimurium, emphasizing the interplay between diet, microbiota, and pathogen susceptibility (45).

Antibiotic-induced disruption of gut microbiota is well-documented, with recovery trajectories varying based on the type of antibiotic, microbiota composition, diet, and other factors (10, 46–50). Streptomycin has been used in various experimental models to investigate the impact of microbiota dysbiosis and its role in colonization resistance (15, 18–26). Our study builds on these findings by highlighting that *B4galnt2*^–/–^ mice exhibit a faster recovery of their microbial communities compared to *B4galnt2*^+/–^ counterparts. Notably, this genotype-specific effect was not consistently observed with kanamycin or vancomycin, suggesting a unique interaction between streptomycin’s microbiota-modulating mechanisms and host genetic factors. These observations reveal the complexity of microbiota recovery, by suggesting a specific interplay between streptomycin-mediated dysbiosis and host genetics, with the intestinal expression of *B4galnt2* being a key factor.

The recovery in *B4galnt2*^–/–^ mice was characterized by an early enrichment of taxa previously associated with colonization resistance or anti-inflammatory roles, including *Enterococcus faecalis*, *Enterocloster clostridioformis*, and members of the *Blautia* genus (34–36, 51, 52). These species, in cooperation with *Klebsiella oxytoca*, are required to confer resistance against the pathogen *Klebsiella pneumoniae* MDR1, by competing for carbon sources (35). Similarly, *Blautia coccoides* and *Enterocloster clostridioformis* together with *E. coli* are important mediators of colonization resistance in *Salmonella* Typhimurium infection in Oligo-Mouse-Microbiota 12 (OMM) (12) mice. In this protective context, these members of the Lachnospiraceae family consume free sugars and play an essential role in protecting against pathogens (52). Although our study did not provide direct glycan profiling or sugar measurements, CAZyme analyses suggest that differences in carbohydrate utilization could contribute to the differences observed in our previous study that performed a streptomycin model of *Salmonella* infection in the same *B4galnt2*-deficient mouse line (19). Genotype-specific patterns also extended to other taxa, such as *Roseburia intestinalis* and *Bifidobacterium longum* which were associated with gut barrier function and immune modulation in previous studies (53–55). *R. intestinalis* modulates immune responses by promoting Treg differentiation and interleukin (IL)−17 secretion, thereby ameliorating colitis in murine models (53, 54). Similarly, *B. longum*, known for its organic acid production and role in reducing intestinal pH, supports recovery and pathogen clearance in conditions like *Clostridioides difficile* infection (55). Although *Akkermansia muciniphila* was not associated with *B4galnt2*^−/−^ mice in our study, its well-characterized barrier-strengthening and pathogen-limiting mechanisms provide a useful conceptual framework for interpreting our findings. *A. muciniphila* promotes gut barrier integrity and antimicrobial peptide secretion, while pasteurized forms of the bacterium enhance resistance to *Salmonella* Typhimurium through inflammasome activation (56). Consistent with these roles, recent work shows that *A. muciniphila* can also protect against *S*. Typhimurium by reducing epithelial adhesion, lowering inflammation, and limiting intestinal fibrosis. While no evidence for direct nutrient competition was shown *in vitro*, *in vivo* effects cannot be excluded (57). Together, these observations suggest that enrichment of barrier-supportive taxa in *B4galnt2^−/−^* mice may contribute to the colonization resistance phenotype previously observed in this genotype (19, 57).

Interestingly, the differential recovery dynamics observed in *B4galnt2*^–/–^ mice may also be influenced by bacterial motility mechanisms and antibiotic resistance profiles. Metatranscriptome analysis reveals a relative increase in flagellin and flagellin-related transcripts in *B4galnt2*^–/–^ mice during recovery, suggesting enhanced motility potential that could contribute to microbial resilience. Swarming motility, characterized by coordinated movement across surfaces, has been linked to alleviation of intestinal inflammation under induced stress conditions (58, 59), and is observed in many bacterial species (60–63). Similarly, surfing motility, which depends on the presence of the mucin glycoprotein, facilitates bacterial navigation and is associated with broad-spectrum antibiotic resistance, including aminoglycosides (64–66). While motility phenotypes were not directly measured, the increased expression of motility-associated genes raises the possibility that enhanced bacterial motility contributed to niche re-establishment during recovery.

The specific analysis of antibiotic resistance genes (ARGs) also reveals important differential transcript abundances between *B4galnt2* genotypes. Notably, the aminoglycoside resistance genes *aadA* and *aadE* are enriched in the early recovery phase of *B4galnt2*^–/–^ mice. Aminoglycoside (3″)(9) adenylyltransferase (*aadA*) adenylates position 3″ of streptomycin and position 9 of spectinomycin, providing resistance to these antibiotics (67, 68). The differential expression of these ARGs underscores the potential functional differences that may contribute to genotype-specific microbiota recovery.

Recent studies indicate that human *B4GALNT2* expression can be variable, driven by allelic variants and epigenetic factors that affect glycotransferase activity and, consequently, the synthesis of glycan structures such as the Sd(a) antigen (69, 70). Such variability in glycosylation may alter the gastrointestinal mucosal environment, potentially influencing microbial colonization and susceptibility to pathogens. Although our findings were obtained in a murine model, they raise the possibility that similar glycosylation-mediated effects could shape microbiota recovery in humans. Importantly, while the observed community-level differences are transient, the immediate post-antibiotic period represents a well-recognized window of increased susceptibility to enteric pathogens in mice. Thus, short-lived shifts during this interval may still hold biological significance. Nevertheless, we caution that the clinical implications remain speculative, as direct evidence linking *B4GALNT2* variation to antibiotic-induced dysbiosis or infection risk in humans is lacking. Future studies should quantify *B4GALNT2* expression across human tissues, define its relationship to microbiota resilience, and determine whether host glycosylation profiles can inform individualized antibiotic or probiotic interventions.

Despite these advances, several limitations of this study should be acknowledged. First, while fecal samples provided valuable insights into microbial dynamics, they may not fully represent microbial communities in other regions of the intestinal tract. In addition, although we identify genotype-associated functional shifts, such as differences in motility-related and antibiotic resistance gene expression, we lack direct mechanistic validation and therefore refrain from causal interpretation. Further experimental validation, particularly regarding motility phenotypes and ARG profiles, will be necessary to determine whether these features actively contribute to recovery dynamics in *B4galnt2*^–/–^ mice. Finally, it is important to note that streptomycin impairs bacterial protein synthesis, including flagellar components. As a result, elevated flagellin transcripts may not directly reflect functional motility during antibiotic exposure, but rather post-treatment recovery processes. Functional assays are needed to validate these interpretations.

In conclusion, our study highlights a significant role of intestinal *B4galnt2* expression in modulating microbiota recovery following streptomycin treatment. The differential recovery pattern observed according to *B4galnt2* genotype draws attention to the importance of host genetic factors in microbial community recovery and suggests that future genotype-specific, microbiota-targeted interventions might mitigate some adverse effects of antibiotic-induced dysbiosis.

## MATERIALS AND METHODS

### Animals and housing

Experiments were conducted at the animal facility of the Max Planck Institute for Evolutionary Biology in Plön, Germany. C57BL/6J *B4galnt2^+/–^* and *B4galnt2^–/–^* mice were raised and housed together as littermates under specific pathogen-free conditions. The mice were kept in open cages and fed with Altromin 1324 standard diet. Cages were changed biweekly, and the light cycle in the mouse room was maintained at 12 h light and 12 h dark.

### Experimental design and antibiotic treatment

Two weeks prior to treatment, *B4galnt2^+/–^* and *B4galnt2^–/–^* mice were grouped into cages according to genotype to be acclimatized for the experiment. Three types of antibiotics, streptomycin (20 mg) (15, 19, 21, 23), kanamycin (10 mg) (16), and vancomycin (40 mg) (17, 30), were administered in a high dose by oral gavage, diluted in 100 μL of water. Control groups for each genotype received 100 µL of water. Thus, a total of eight groups of mice, with *n* = 7 each, were analyzed. Fecal samples were collected before and after antibiotic treatment on the following days: −4, 0, 1, 2, 3, 4, 10, and 15 (Fig. 1). Both male and female mice were included in all experimental groups, and sex was incorporated as a covariate in statistical analyses. Detailed metadata including sex, genotype, and antibiotic treatment for each individual mouse are provided in Table S6. To minimize the influence of circadian rhythms on host and microbiota, fecal samples were collected directly from individual mice consistently between 10:00 a.m. and 12:00 p.m. on any given collection day and immediately placed into RNAlater solution (Thermo Fisher Scientific).

### Simultaneous DNA and RNA extraction, 16S rRNA gene (DNA) and transcript (RNA) amplification, and amplicon sequencing

Simultaneous extraction of DNA and RNA from fecal pellets was performed using the Qiagen AllPrep DNA/RNA Kit (Qiagen, Hilden, Germany). Samples were homogenized in 600 μL of RLT buffer (Qiagen) with a Lysing Matrix E tubes (MPBio) and Precellys (3 × 15 s). DNase I Solution (Stemcell technologies) was used to treat the RNA extracts and remove any DNA contamination. The cDNA was synthesized from RNA using the High-Capacity cDNA Reverse Transcription kit (Thermo Fisher Scientific), following the manufacturer’s instructions.

The hypervariable region V1-V2 of the 16S rRNA gene was amplified using universal bacterial primers 27F (5′-AGAGTTTGATCCTGGCTCAG-3′) and 338R (5′-TGCTGCCTCCCGTAGGAGT-3′) (71). PCR amplification was carried out using the following protocol: an initial denaturation at 98°C for 3 min, followed by 30 cycles consisting of denaturation at 98°C for 9 s, annealing at 55°C for 1 min, and extension at 72°C for 1 min, with a final extension step at 72°C for 10 min. The resulting PCR products were quantified using a GelDoc XR+ (BioRad) and pooled in equimolar proportions. The ZymoBIOMICS Microbial Community DNA Standard containing 8 bacterial and 2 fungal species was amplified and used as a sequencing quality control. Libraries were sequenced on the Illumina MiSeq platform using the v2 kit (2 × 250 bp).

### 16S rRNA gene (DNA) and transcript (RNA) processing and analysis

Raw reads were demultiplexed using bcl2fastq, allowing no mismatches in the barcodes. Subsequent analyses, including quality filtering, denoising, chimera removal, taxonomic assignment, and phylogenetic tree construction, were performed within the Qiime2 framework (version 2023.05) (72). Briefly, denoising, chimera removal, and identification of representative sequences were performed using DADA2 (73). Taxonomic annotation of representative sequences for amplicon sequence variants (ASVs) was accomplished utilizing the SILVA database (v 138) and a pre-trained naive Bayes classifier (74, 75). The ASV sequences were used to construct a tree for phylogenetic diversity analyses. Prior to tree construction, a multiple sequence alignment was generated using MAFFT (76), after which all highly variable and uninformative alignment columns were masked. The unrooted phylogenetic tree was constructed using FastTree2 (77) from the masked alignments. The tree was subsequently rooted at the midpoint of the longest tip-to-tip distance in the unrooted tree.

The resulting ASV feature table, phylogenetic tree, and reference sequences from Qiime2 were imported into R v4.2.2 along with the metadata table using the qiime2R v0.99.6 and phyloseq v1.42.0 packages (78, 79). ASVs observed in only one sample were removed. Rarefaction curve analysis was used to estimate the completeness of microbial sampling, and a depth of 4,500 sequences was selected (Fig. S2).

### Metagenomics and metatranscriptomics

Samples from the streptomycin treatment group were selected for metagenomic and metatranscriptomic analysis based on longitudinal recovery patterns, as assessed via 16S rRNA gene profiling. These time points include before treatment (day 0), immediately after treatment (day 1), and during early (day 3) and late (day 15) recovery phases (Fig. 1).

Metagenomic sequencing libraries were constructed using NexteraXT technology, as described by the manufacturer (Illumina). The libraries were sequenced using the Illumina NextSeq 550 System High-Output kit.

For selected RNA samples, ribosomal RNA depletion was performed using the QIAseq FastSelect −5S/16S/23S Kit (Qiagen), and the Illumina TruSeq protocol was used to prepare the RNA-Seq library. The 56 streptomycin group RNA samples were sequenced on the Illumina NovaSeq platform with 100 bp single-end reads.

Metagenomic and metatranscriptomic sequencing data were preprocessed using KneadData (v0.7.10). KneadData includes FastQC (v0.12.1) for quality assessment, Trimmomatic (v0.39) (80) for quality filtering, and Bowtie2 (v1.2.2) (81) for host sequence decontamination.

Metagenomic taxonomic profiles were generated using Kraken v2.1.2 (confidence level 0.05) and Bracken v2.2 with the PlusPFP database, which includes bacterial, archaeal, viral, plasmid, fungal, protozoan, and plant indices (82, 83). The resulting microbiota abundances and associated metadata tables were imported into R using the biomformat v1.26.0 and phyloseq v1.42.0 packages (79, 84). Samples with at least 500,000 Kraken2/Bracken classified reads were kept for taxonomic analysis. Taxa with an average abundance of ≥0.0025% were retained for downstream analyses.

The HMP Unified Metabolic Analysis Network 3 (HUMAnN3 v3.6) pipeline was used to generate functional profiles for both metagenome and metatranscriptome data sets (42). Functional annotations were stratified to KEGG Orthology (KO) groups for pathway-based analysis. In parallel, Gene Ontology (GO) term profiles were generated to enable functional enrichment analyses at the level of biological processes. For GO-based analyses, we retained terms with a minimum average abundance of 100 counts per million (CPM) per timepoint to reduce noise from low-abundance features and improve robustness of downstream comparisons.

### Antibiotic resistance genes abundance profiling

Short reads were queried for antibiotic resistance genes (ARG) using DeepARG (v1.0.2) with default settings (identity cutoff for sequence alignment—50, *E*-value cutoff—1*e*-10, alignment read overlap—0.8) (85). ARG abundances were normalized to relative abundance, and features were retained for downstream analysis only if they reached a minimum abundance of 0.0001% in at least one timepoint.

### Carbohydrate active enzyme CAZyme abundance profiling

Short reads were queried for the presence of CAZyme encoding genes using the newly developed Cayman (86) package (v0.10.1). We used MGX and MTX reads as single end in the mapping procedure with default settings in the “profile” workflow. As target databases, we employed all provided gene catalogs to detect a maximum of CAZyme genes (soil, marine, freshwater, wastewater, human-vagina/skin/oral/nose/gut, pig-gut, mouse-gut, dog-gut, cat-gut) (86). The Gene catalogs were based on nonredundant and prevalence filtered 95% ID clustered versions of the catalogs published by (87) (GMGC v1.0) (87).

### Microbial community analysis

Alpha diversity was assessed using ASV richness, the Shannon diversity index, and Faith’s phylogenetic diversity (88) for each *B4galnt2* genotype and time point. The Mann–Whitney–Wilcoxon test was applied to compare groups.

Genotype-specific trajectories of alpha diversity were assessed using linear mixed-effects models implemented in the nlme R package (v3.1). For each diversity metric (species richness, Shannon diversity, Simpson evenness estimated from metagenomic profiles), the model included fixed effects of genotype, time, and their interaction. Time was modeled as a categorical factor (with day 0 as baseline), and an AR(1) correlation structure with numeric sampling day was specified to account for within-mouse temporal autocorrelation. Mouse ID was included as a random effect to account for repeated measures. Likelihood ratio tests (LRTs) compared models with and without the genotype × time interaction. Estimated marginal means (EMMs) were computed using the emmeans package, with Holm-adjusted contrasts to test between-genotype differences at each time point and within-genotype differences relative to baseline.

To explore the community structure and to visualize the clustering of samples based on their compositional similarities, principal coordinate analysis (PCoA) was performed using the phyloseq v1.42.0 package (79). Beta diversity was assessed using PERMANOVA as implemented in the vegan package in R with 9,999 permutations on Bray–Curtis and weighted UniFrac distance matrices (89). To account for covariates, models were specified as follows: comparisons between genotypes at individual timepoints were tested using sex + genotype + genotype:sex; comparisons between treatment and control groups used sex + treatment_group + treatment_group:sex; and comparisons between treatment groups at post-treatment timepoints and their corresponding baseline samples used sex + time + time:sex.

Microbiome Multivariable Associations with Linear Models (MaAsLin2; v1.15.1) was used to quantify differences in taxonomic, functional, and antimicrobial resistance gene (ARG) profiles by *B4galnt2* genotype, with sex modeled as a fixed effect (33). All MaAsLin2 analyses were performed on data sets stratified by timepoint.

Statistical analyses were conducted in R (v4.2.2) using the rstatix package (v0.7.2). Graphs were generated with ggplot2 (v3.4.4) (90). Spearman correlation was utilized to examine associations between continuous variables. The Mann–Whitney–Wilcoxon test (91, 92) was employed to test for differences in means of continuous variables. Wilcoxon effect sizes were also calculated, with the effect size (*r*) defined as the *Z* statistic divided by the square root of the sample size (*n*). Correction for multiple testing was performed using the false discovery rate (FDR) method for each treatment, group, or time point when appropriate (93).

For statistical analyses, we used the normalized combined abundances, normalized via “reads per kilobase of transcript per million mapped reads” (RPKM). CAZyme profiles were compared and structured via nonmetric multidimensional scaling of Bray-Curtis dissimilarities in vegan (v2.7-1) and analyzed via permutational multivariate analyses of variance (PERMANOVA) as implemented via distance based redundancy analyses (capscale) using 10,000 permutations to derive *P*-values. To analyze the abundance changes of CAZymes over time, we employed linear mixed-effects models investigating the interaction of genotypes and time after streptomycin treatment. These models were implemented via lmer (lme4 v1.1-37) with mouse ID included as a random factor. Significance was assessed with lmerTest (v3.1-3), and conditional and marginal *R*² values were derived via MuMIn (r.squaredGLMM, v1.48.11). We selected only CAZymes which showed a significant interaction and thus *B4galnt2* genotype specific change over the time course. Single time points were tested via Wilcoxon signed rank tests.

### Network analysis

To construct microbial correlation-based networks, we analyzed the 16S rRNA gene sequence data using the NetCoMi R package (94). Separate networks were created for the different data sets (DNA, RNA), groups (Ctrl, STR, KAN, and VAN), genotypes, and timepoints. Microbial genera act as nodes, with edges representing Pearson correlation coefficients between pairs of genera. Edges with an absolute Pearson correlation >0.5 were kept. Data normalization was performed using the centered log-ratio (clr) transformation, and zeros were replaced using the multiplicative simple replacement method (R package zCompositions, function“multRepl”) prior to pairwise Pearson correlation analysis (95). Networks were further analyzed by calculating global network characteristics.

## Data Availability

The original contributions presented in the study are publicly available. These data can be found here: https://www.ebi.ac.uk/ena under the Project accession number PRJEB100772.

## SUPPLEMENTAL MATERIAL

Supplemental material msystems.01640-25-s0001.pdf Supplemental figures and tables.

ASM does not own the copyrights to Supplemental Material that may be linked to, or accessed through, an article. The authors have granted ASM a non-exclusive, world-wide license to publish the Supplemental Material files. Please contact the corresponding author directly for reuse.
