# Supplementary material for: Host genetics shapes the recovery of the gut microbiome after antibiotic treatment: the role of the blood group related B4galnt2 gene
Source: mSystems. 2026 Apr 30;11(5):e01640-25. doi: 10.1128/msystems.01640-25 (PMC13185590; doi:10.1128/msystems.01640-25)
Supplement: Supplemental material — Supplemental figures and tables. [file msystems.01640-25-s0001.pdf]

Supplementary data

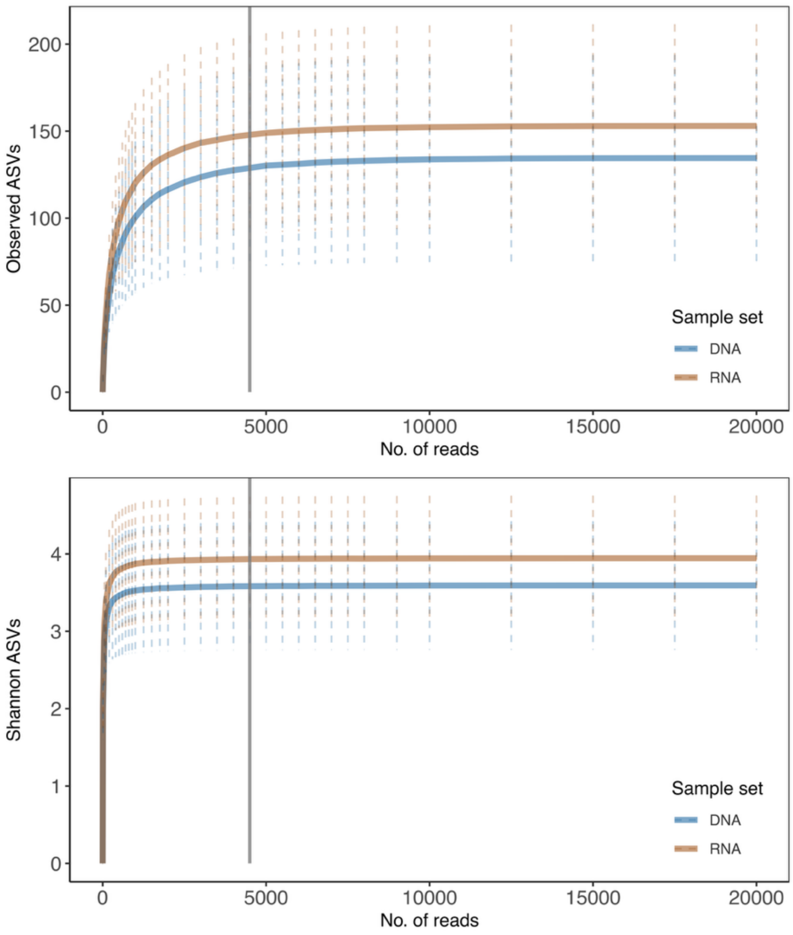

Supplementary Figure 1: Rarefaction curves based on number of observed ASVs, and Shannon index.

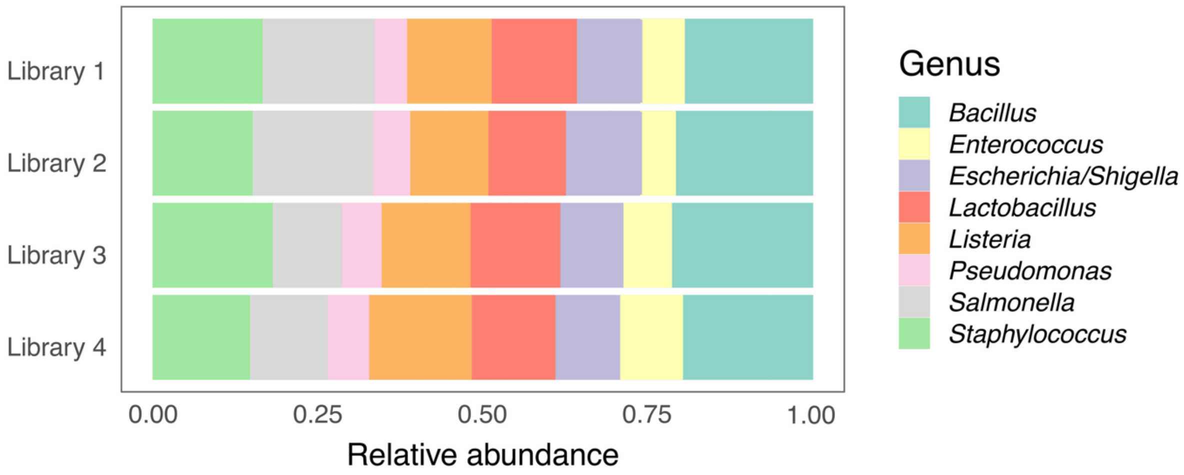

Supplementary Figure 2: Analysis of a mock community as a positive sequencing control.

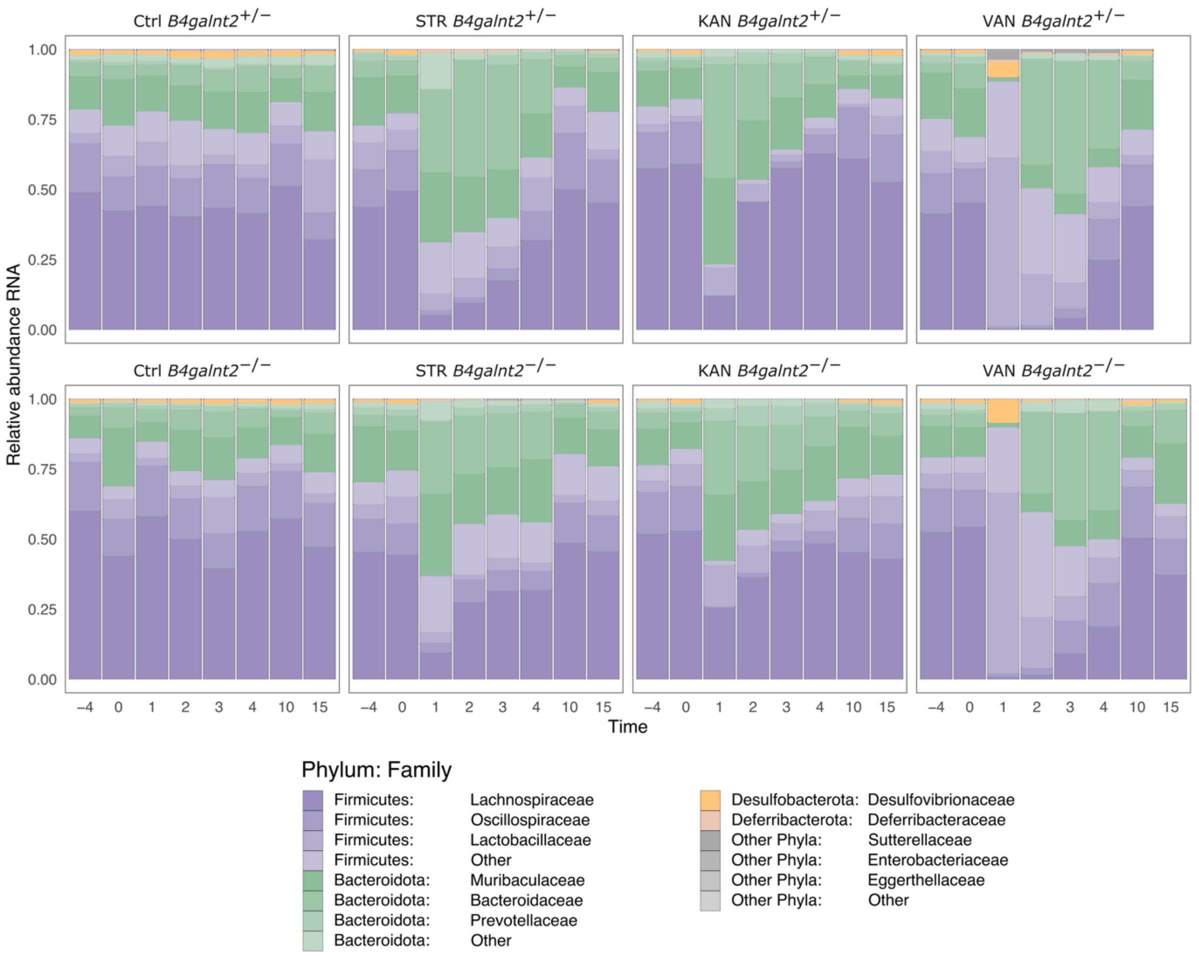

Supplementary Figure 3: Fecal microbiome composition dynamics based on 16S rRNA transcript (RNA) sequencing: Relative sequence abundance in *B4galnt2*-associated microbial communities at the phylum and family levels; bars represent the mean relative abundance of taxa across all available mice within each genotype and timepoint

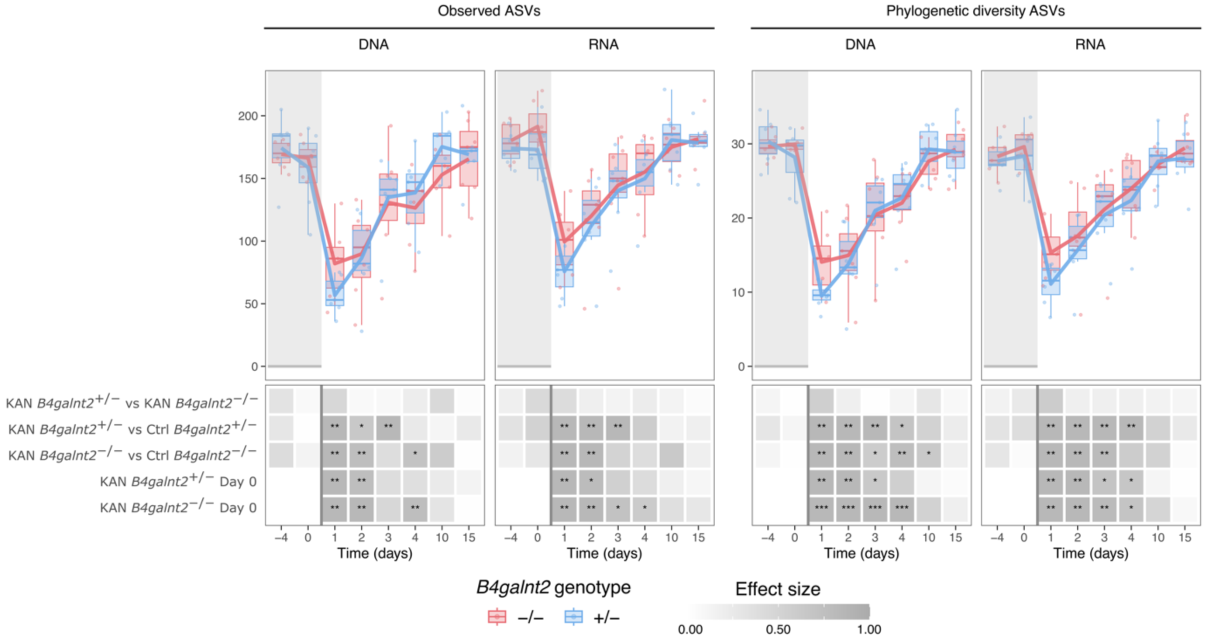

Supplementary Figure 4: ASV richness and phylogenetic diversity (PD) in the kanamycin treated mice at the 16S rRNA gene (DNA) and transcript (RNA) levels. Stars denote significance: *$p_{adj} < 0.05$, **$p_{adj} < 0.01$, ***$p_{adj} < 0.001$

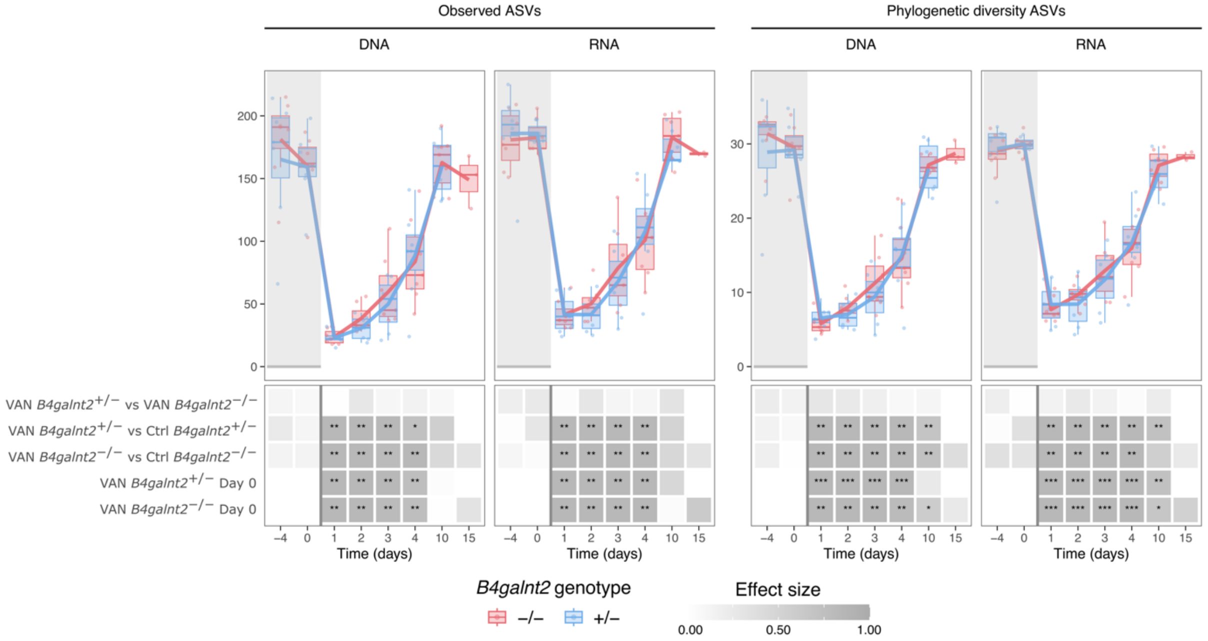

Supplementary Figure 5: ASV richness and phylogenetic diversity (PD) in the vancomycin treated mice at the 16S rRNA gene (DNA) and transcript (RNA) levels. Stars denote significance: *$p_{adj} < 0.05$, **$p_{adj} < 0.01$, ***$p_{adj} < 0.001$

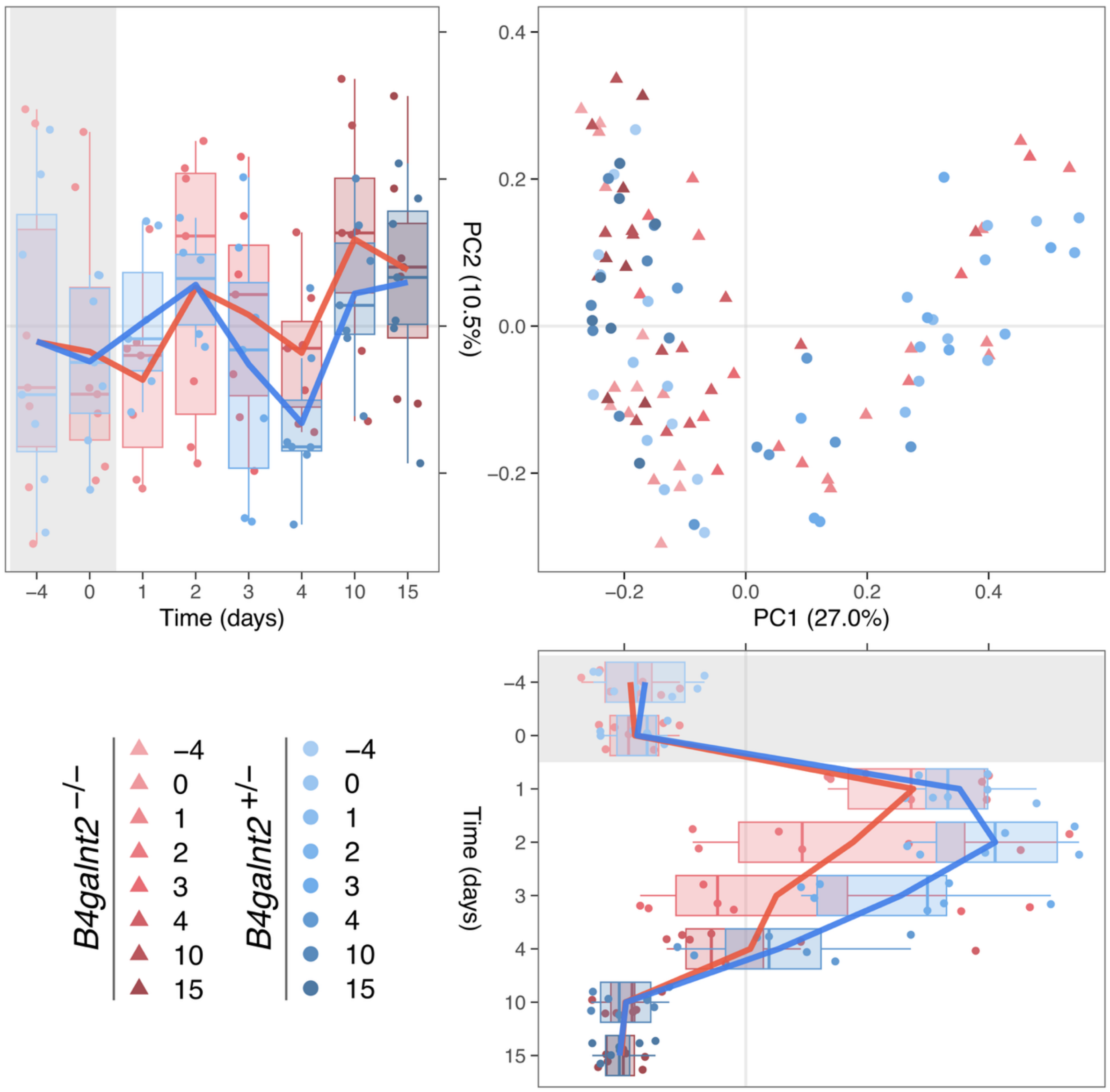

Supplementary Figure 6: Principal Coordinates Analysis (PCoA) plot of samples before, during and after the streptomycin treatment based on Bray-Curtis distances at the 16S rRNA gene (DNA) level. The distribution of samples by *B4galnt2* groups and time is shown along the first and second axes of the PCoA plot

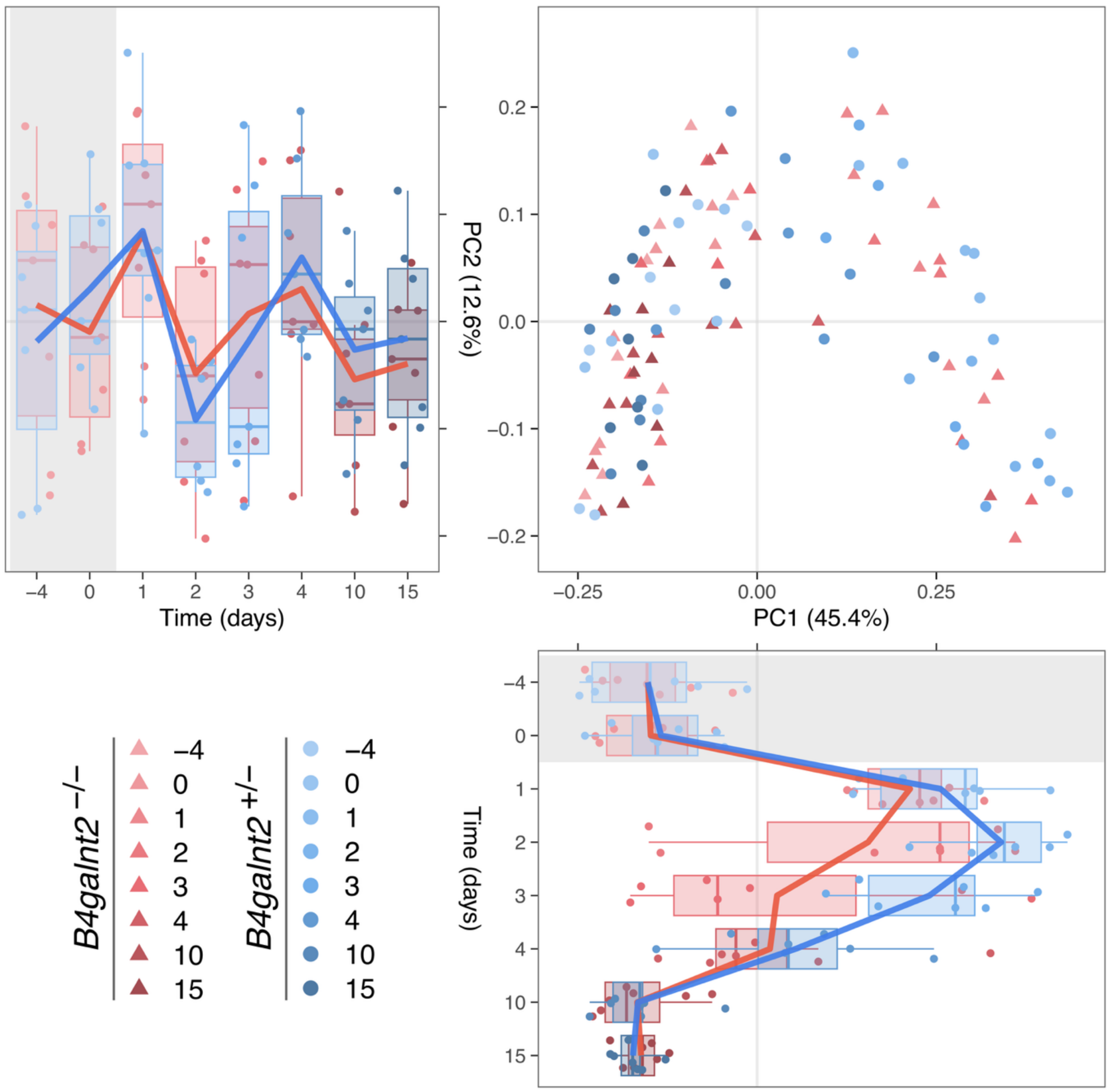

Supplementary Figure 7: Principal Coordinates Analysis (PCoA) plot of samples before, during and after the streptomycin treatment based on weighted Unifrac (W-UniFrac) distances at the 16S rRNA gene (DNA) level. The distribution of samples by *B4galnt2* groups and time is shown along the first and second axes of the PCoA plot

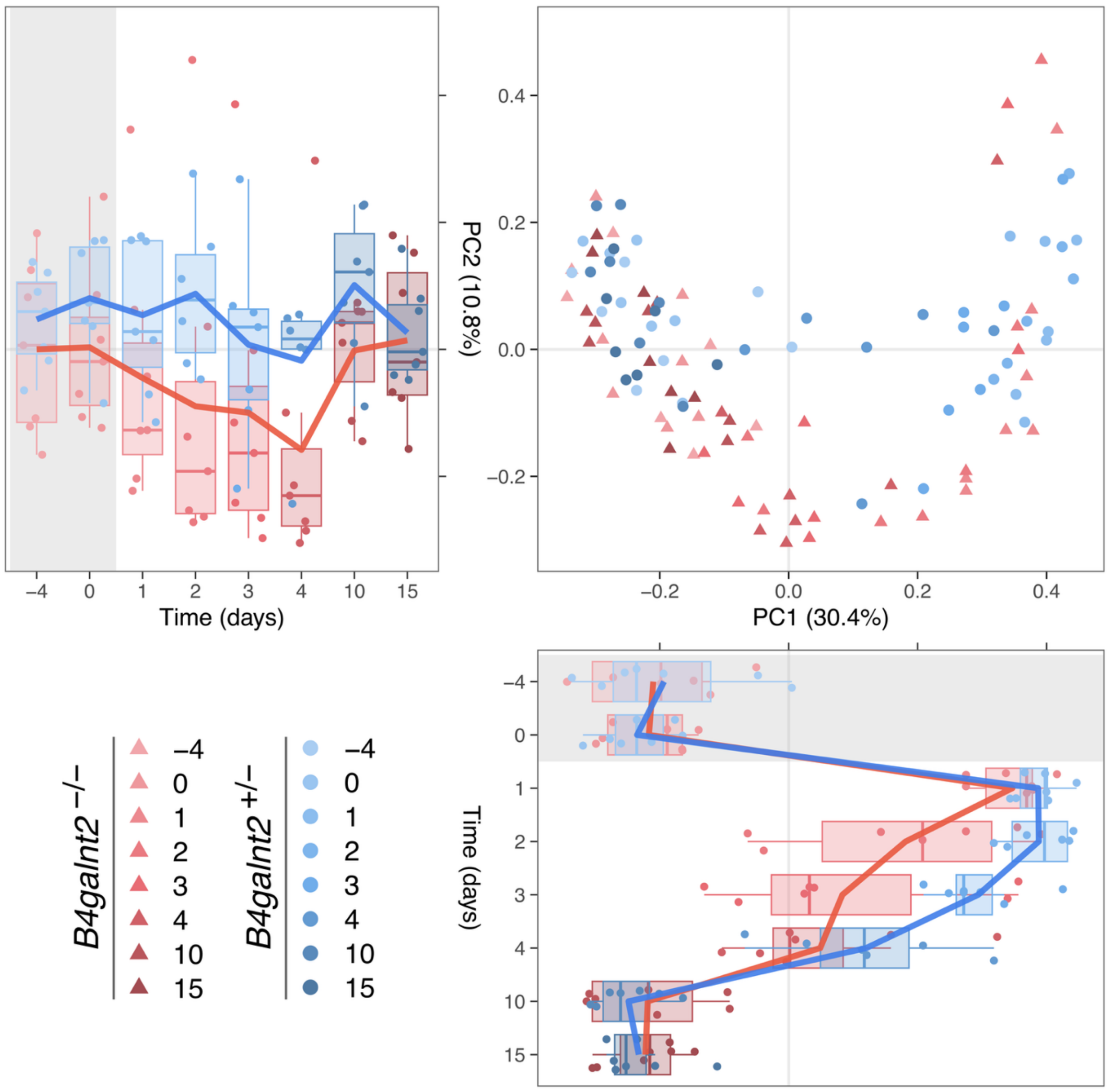

Supplementary Figure 8: Principal Coordinates Analysis (PCoA) plot of samples before, during and after the streptomycin treatment based on Bray-Curtis distances at the 16S rRNA transcript (RNA) level. The distribution of samples by *B4galnt2* groups and time is shown along the first and second axes of the PCoA plot

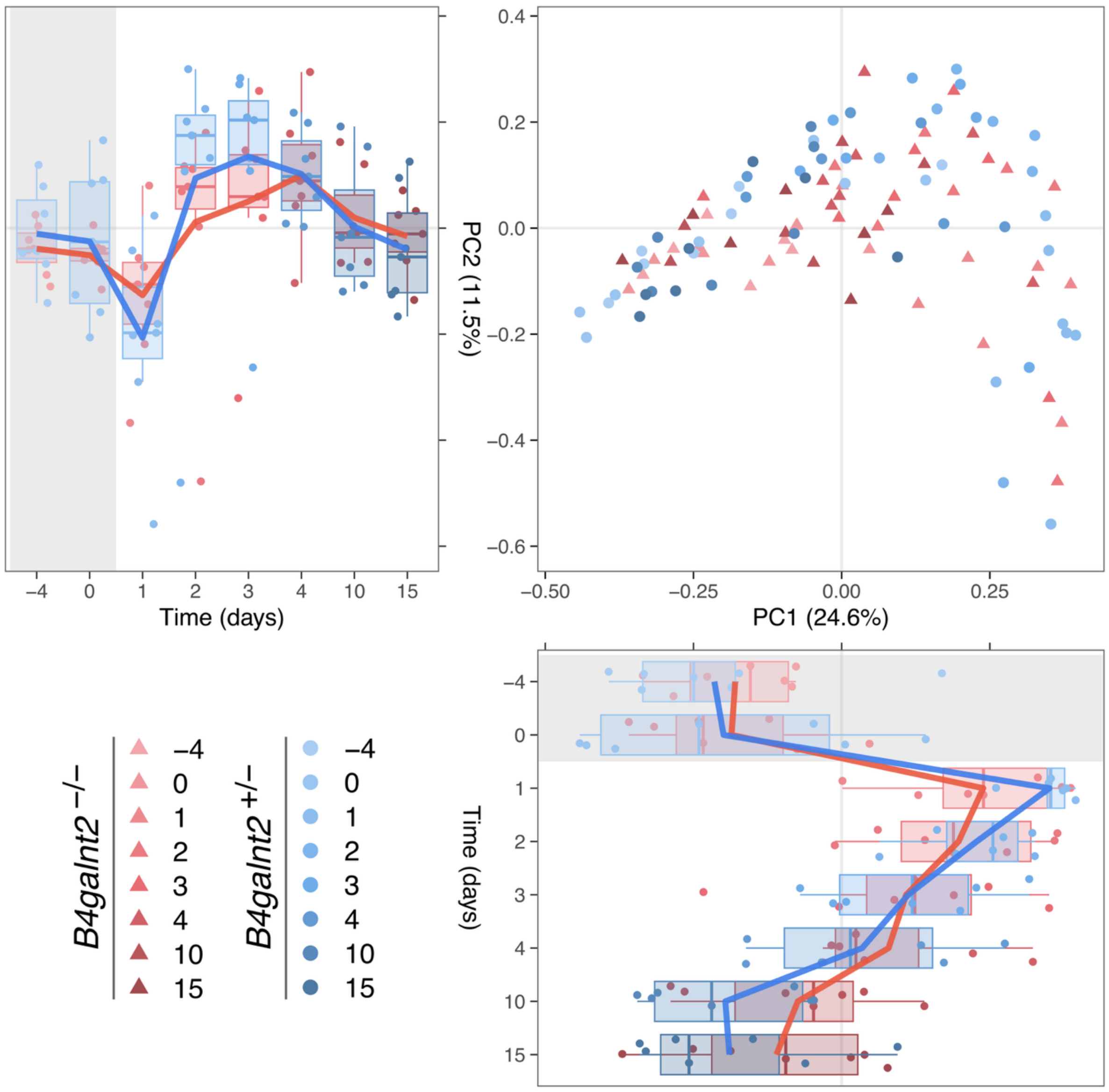

Supplementary Figure 9: Principal Coordinates Analysis (PCoA) plot of samples before, during and after the kanamycin treatment based on Bray-Curtis distances at the 16S rRNA gene (DNA) level. The distribution of samples by *B4galnt2* groups and time is shown along the first and second axes of the PCoA plot

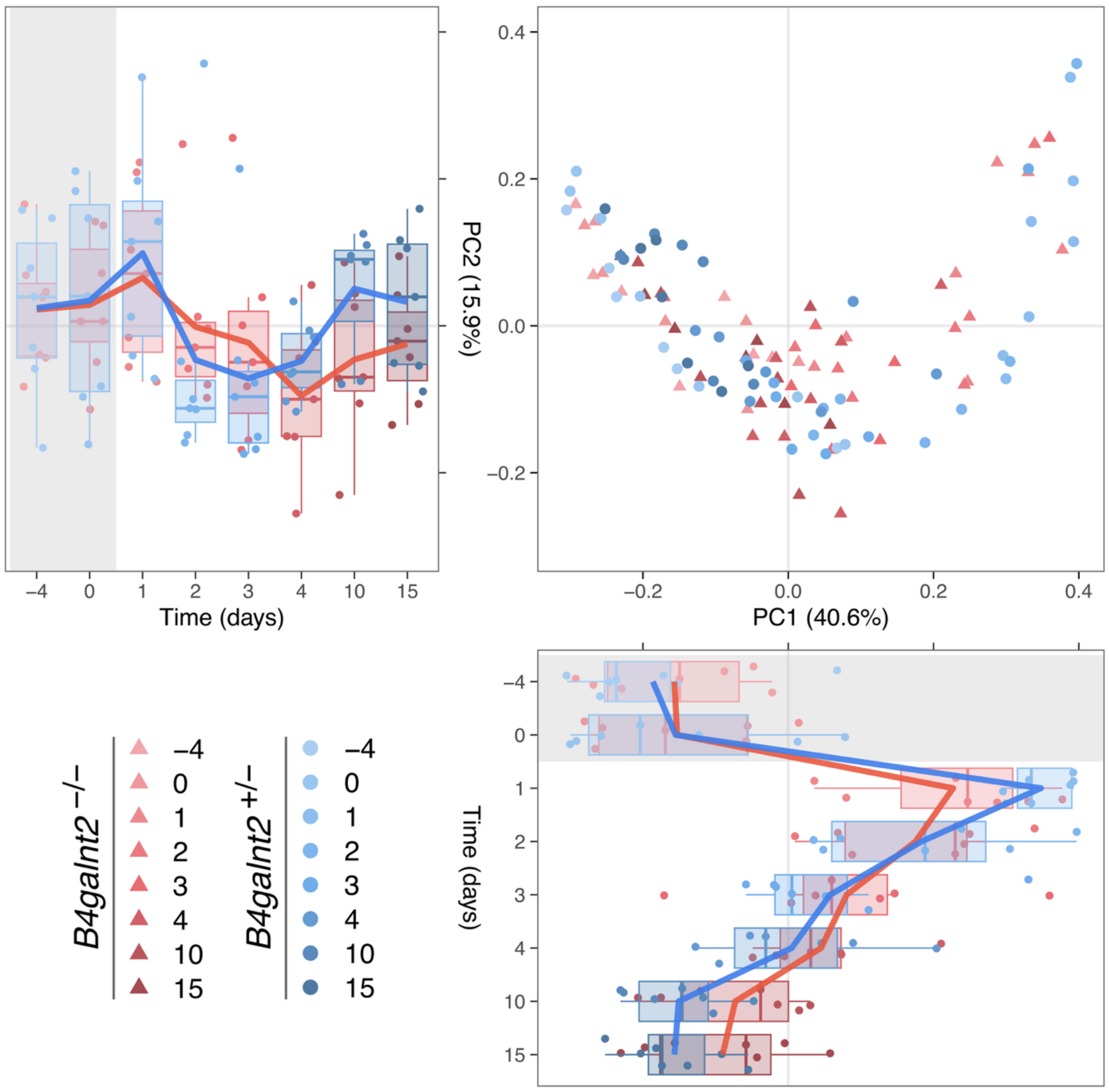

Supplementary Figure 10: Principal Coordinates Analysis (PCoA) plot of samples before, during and after the kanamycin treatment based on weighted Unifrac (W-UniFrac) distances at the 16S rRNA gene (DNA) level. The distribution of samples by *B4galnt2* groups and time is shown along the first and second axes of the PCoA plot

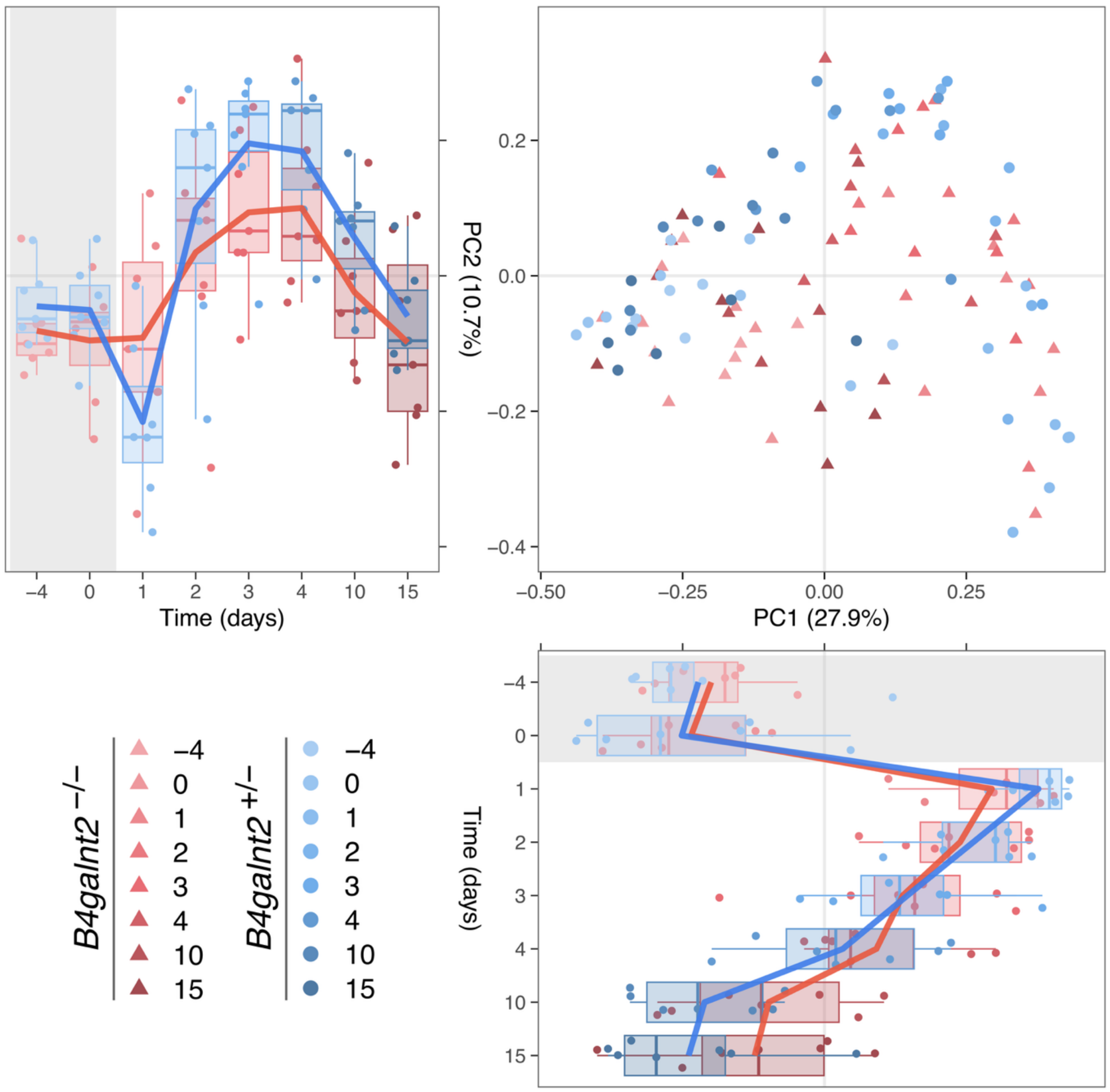

Supplementary Figure 11: Principal Coordinates Analysis (PCoA) plot of samples before, during and after the kanamycin treatment based on Bray-Curtis distances at the 16S rRNA transcript (RNA) level. The distribution of samples by *B4galnt2* groups and time is shown along the first and second axes of the PCoA plot

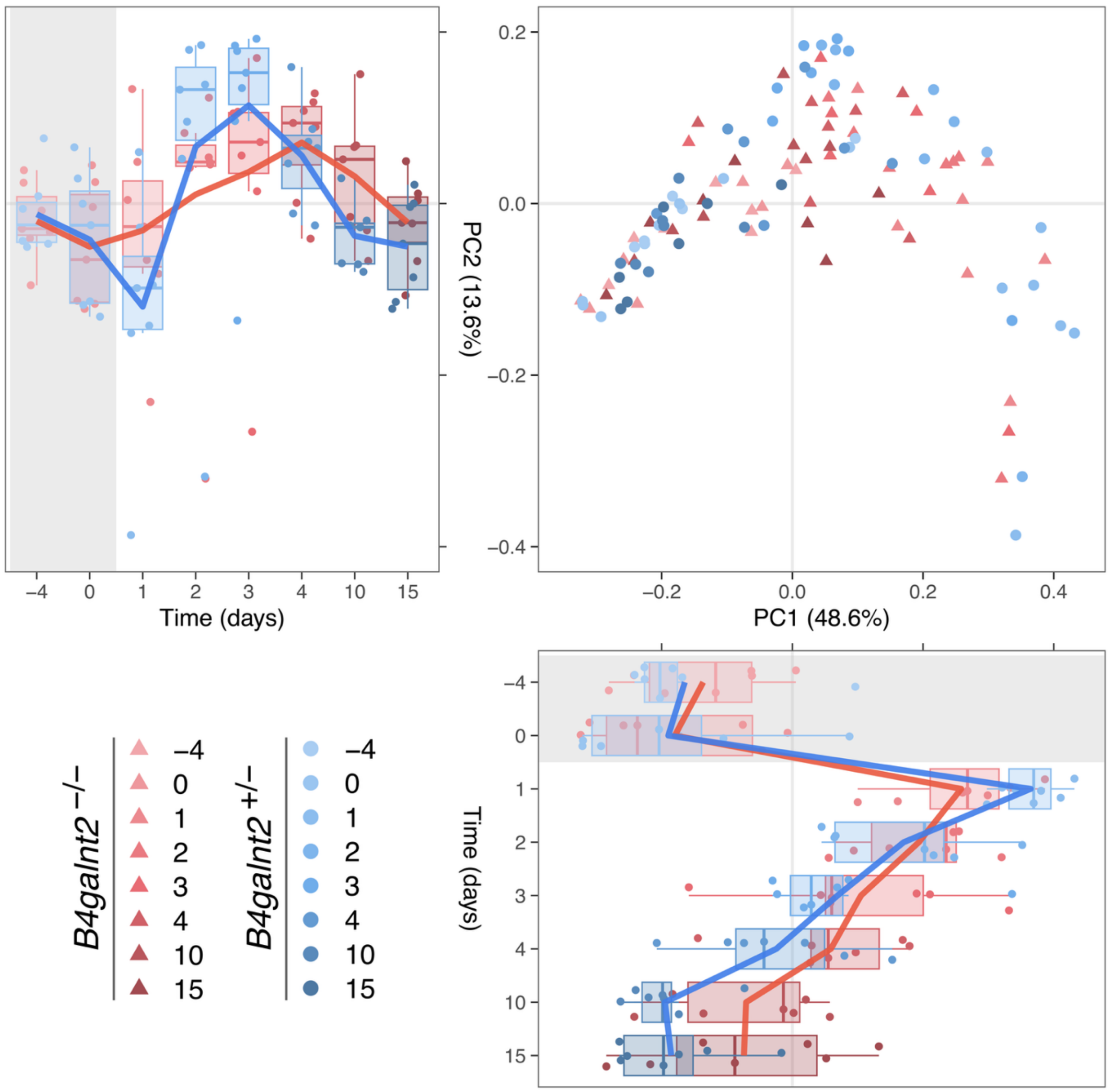

Supplementary Figure 12: Principal Coordinates Analysis (PCoA) plot of samples before, during and after the kanamycin treatment based on weighted Unifrac (W-UniFrac) distances at the 16S rRNA transcript (RNA) level. The distribution of samples by *B4galnt2* groups and time is shown along the first and second axes of the PCoA plot

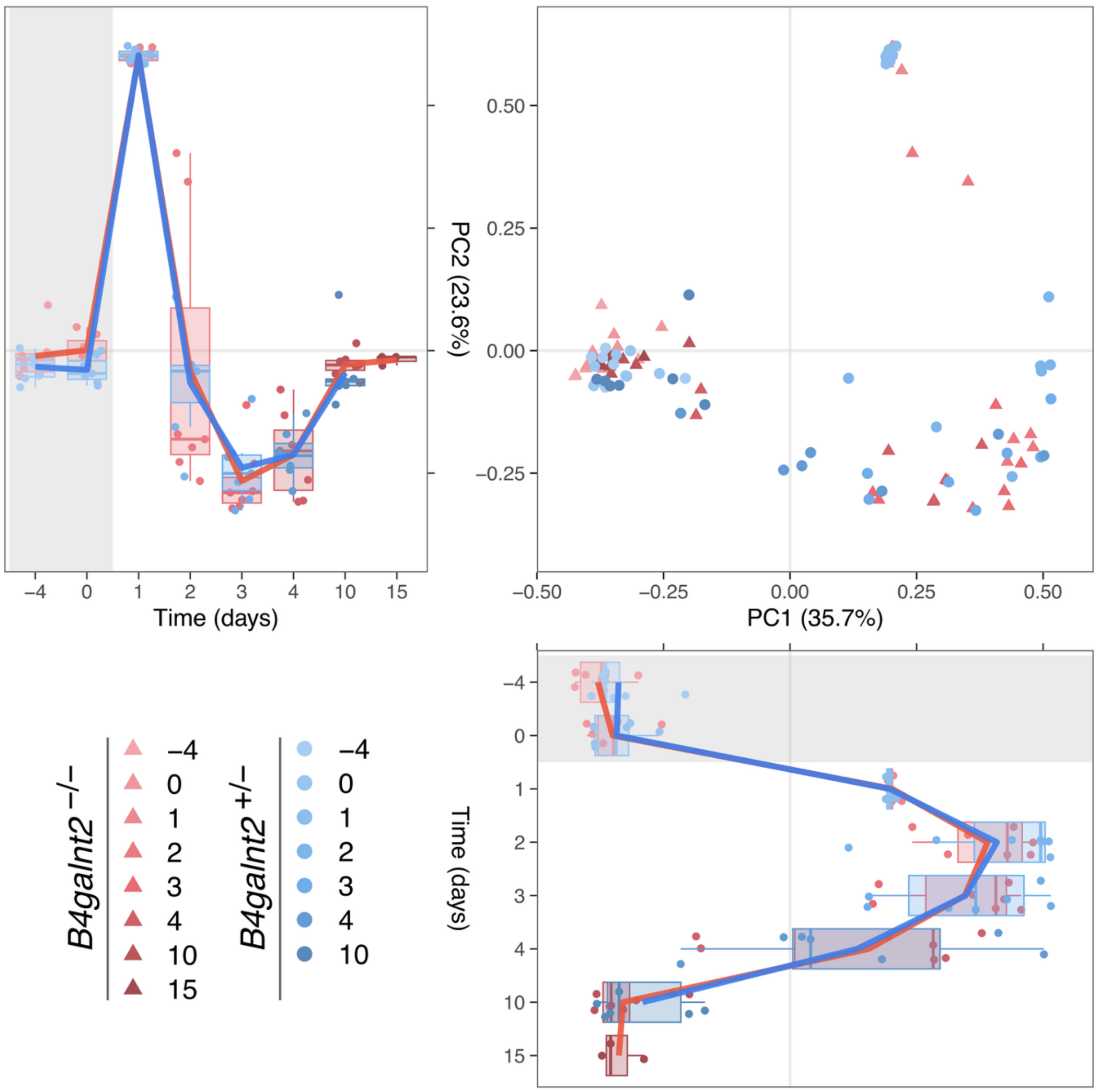

Supplementary Figure 13: Principal Coordinates Analysis (PCoA) plot of samples before, during and after the vancomycin treatment based on Bray-Curtis distances at the 16S rRNA gene (DNA) level. The distribution of samples by *B4galnt2* groups and time is shown along the first and second axes of the PCoA plot

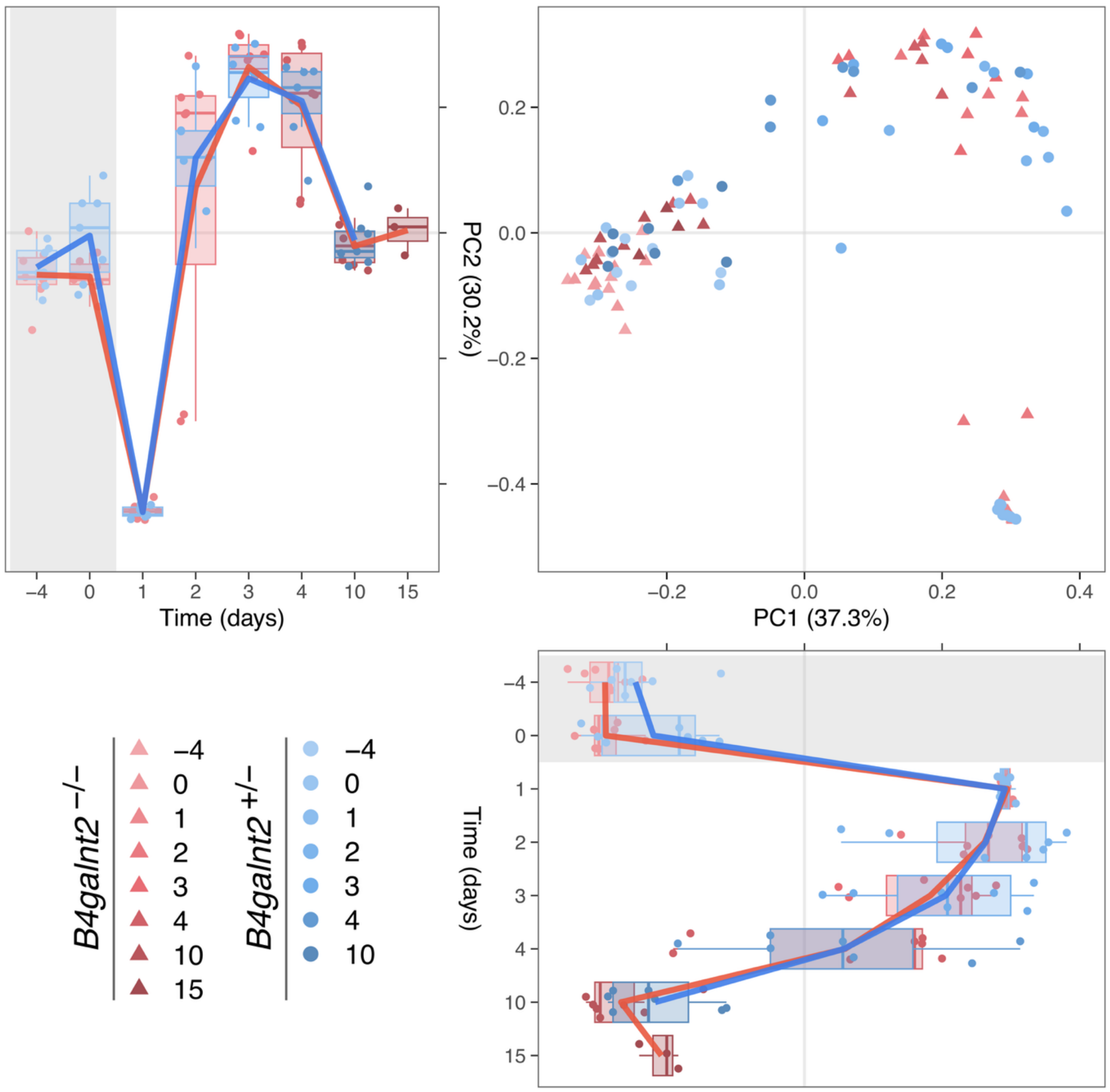

Supplementary Figure 14: Principal Coordinates Analysis (PCoA) plot of samples before, during and after the vancomycin treatment based on weighted Unifrac (W-UniFrac) distances at the 16S rRNA gene (DNA) level. The distribution of samples by *B4galnt2* groups and time is shown along the first and second axes of the PCoA plot

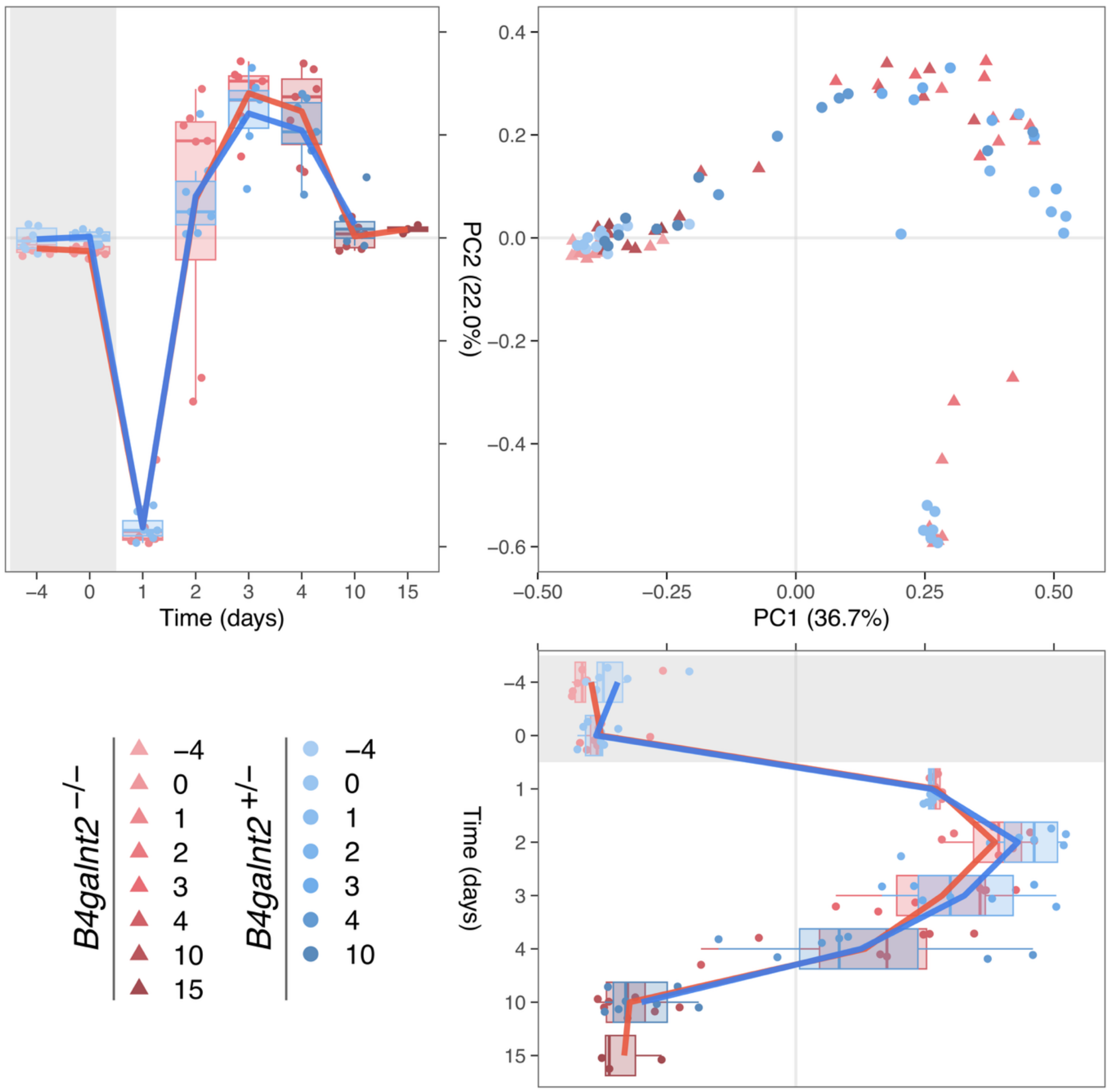

Supplementary Figure 15: Principal Coordinates Analysis (PCoA) plot of samples before, during and after the vancomycin treatment based on Bray-Curtis distances at the 16S rRNA transcript (RNA) level. The distribution of samples by *B4galnt2* groups and time is shown along the first and second axes of the PCoA plot

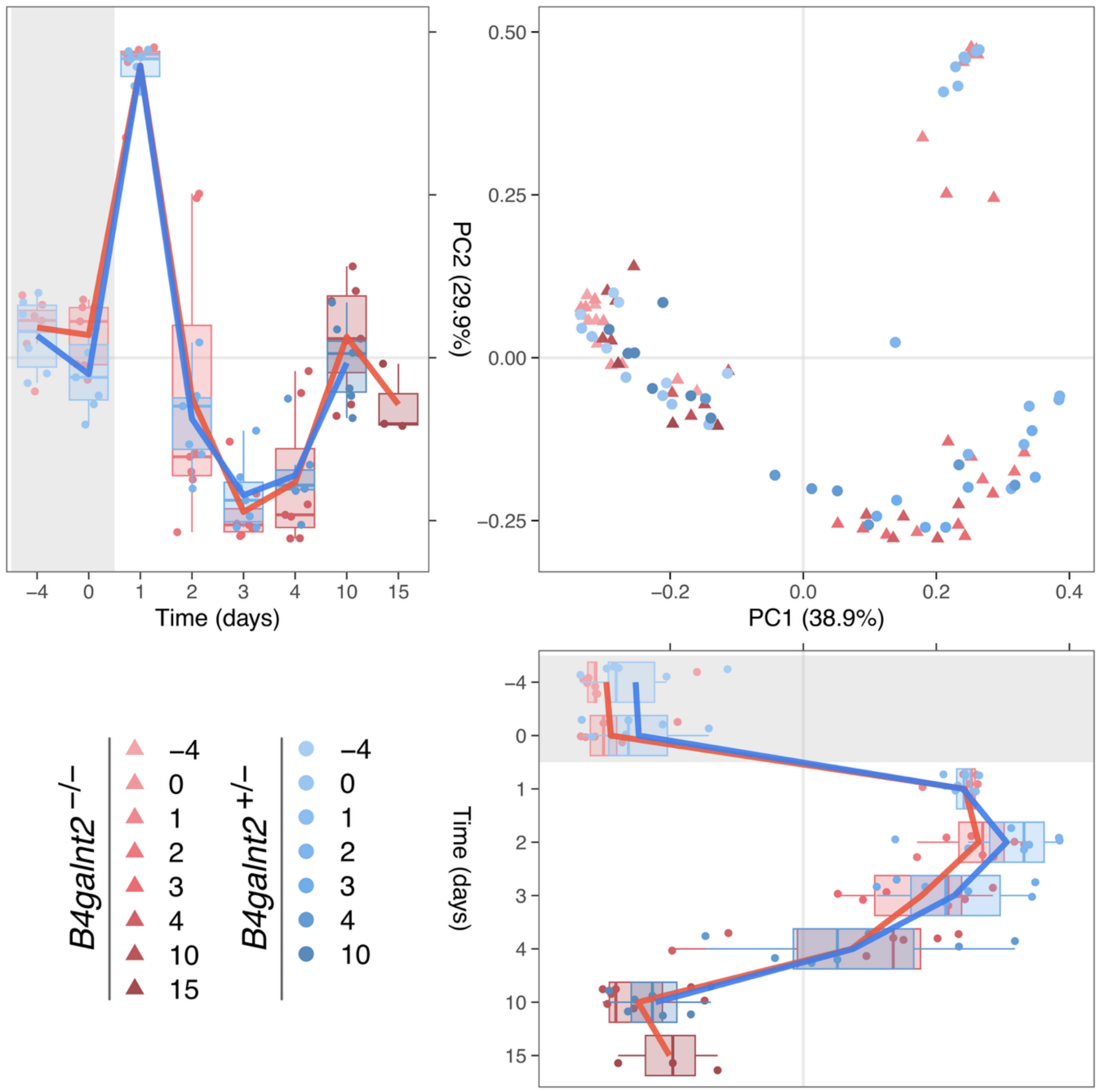

Supplementary Figure 16: Principal Coordinates Analysis (PCoA) plot of samples before, during and after the vancomycin treatment based on weighted Unifrac (W-UniFrac) distances at the 16S rRNA transcript (RNA) level. The distribution of samples by *B4galnt2* groups and time is shown along the first and second axes of the PCoA plot

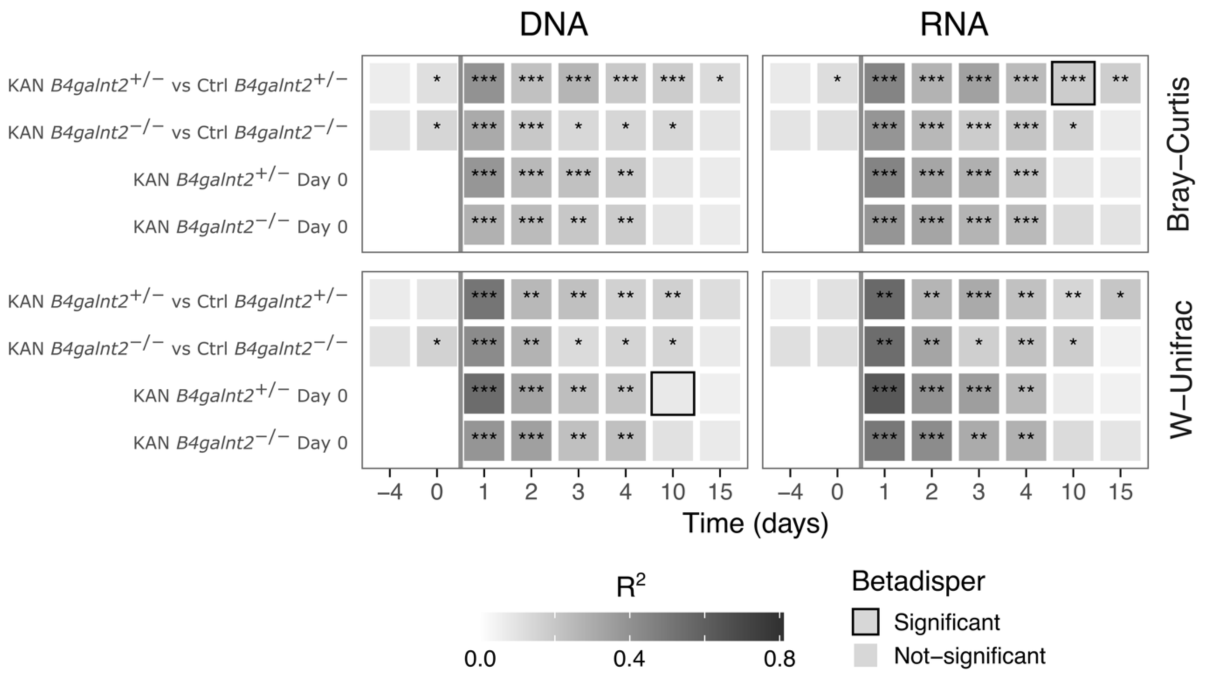

Supplementary Figure 17: Significance and effect size estimates for PERMANOVA and Betadisper analysis for differences in the kanamycin treated mice at 16S rRNA gene (DNA) and transcript (RNA) levels between *B4galnt2* genotypes. Stars denote significance: *$p_{adj} < 0.05$, **$p_{adj} < 0.01$, ***$p_{adj} < 0.001$.

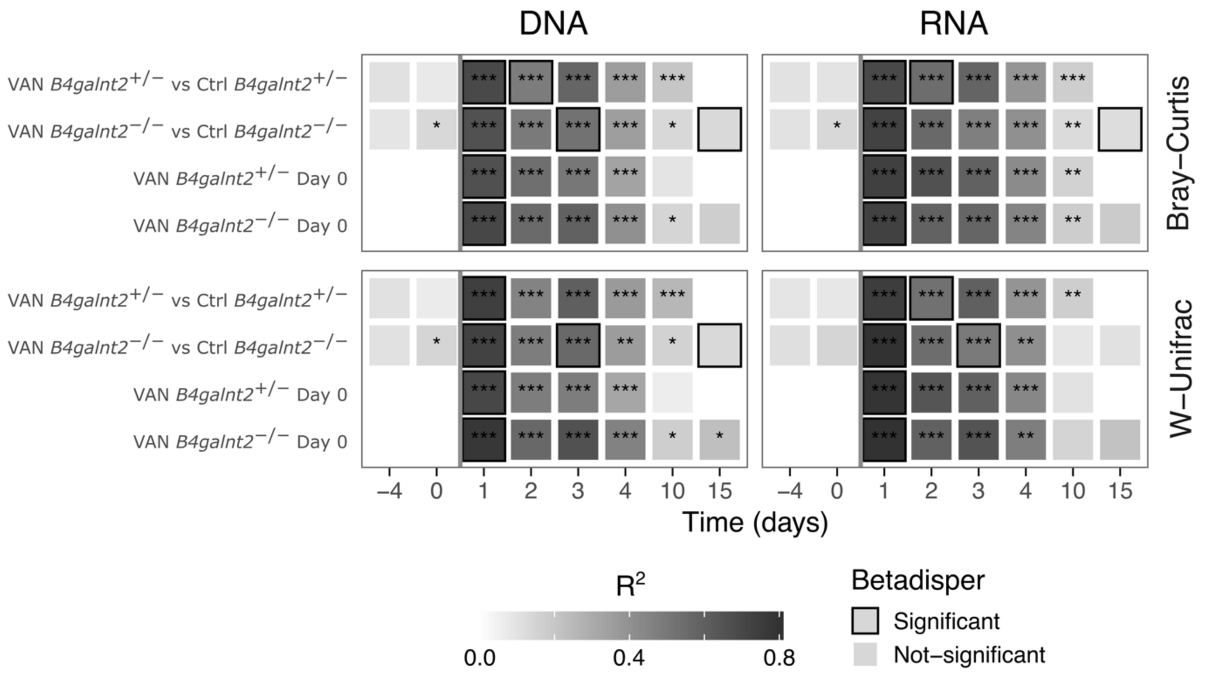

Supplementary Figure 18: Significance and effect size estimates for PERMANOVA and Betadisper analysis for differences in the vancomycin treated mice at 16S rRNA gene (DNA) and transcript (RNA) levels between *B4galnt2* genotypes. Stars denote significance: *$p_{adj} < 0.05$, **$p_{adj} < 0.01$, ***$p_{adj} < 0.001$.

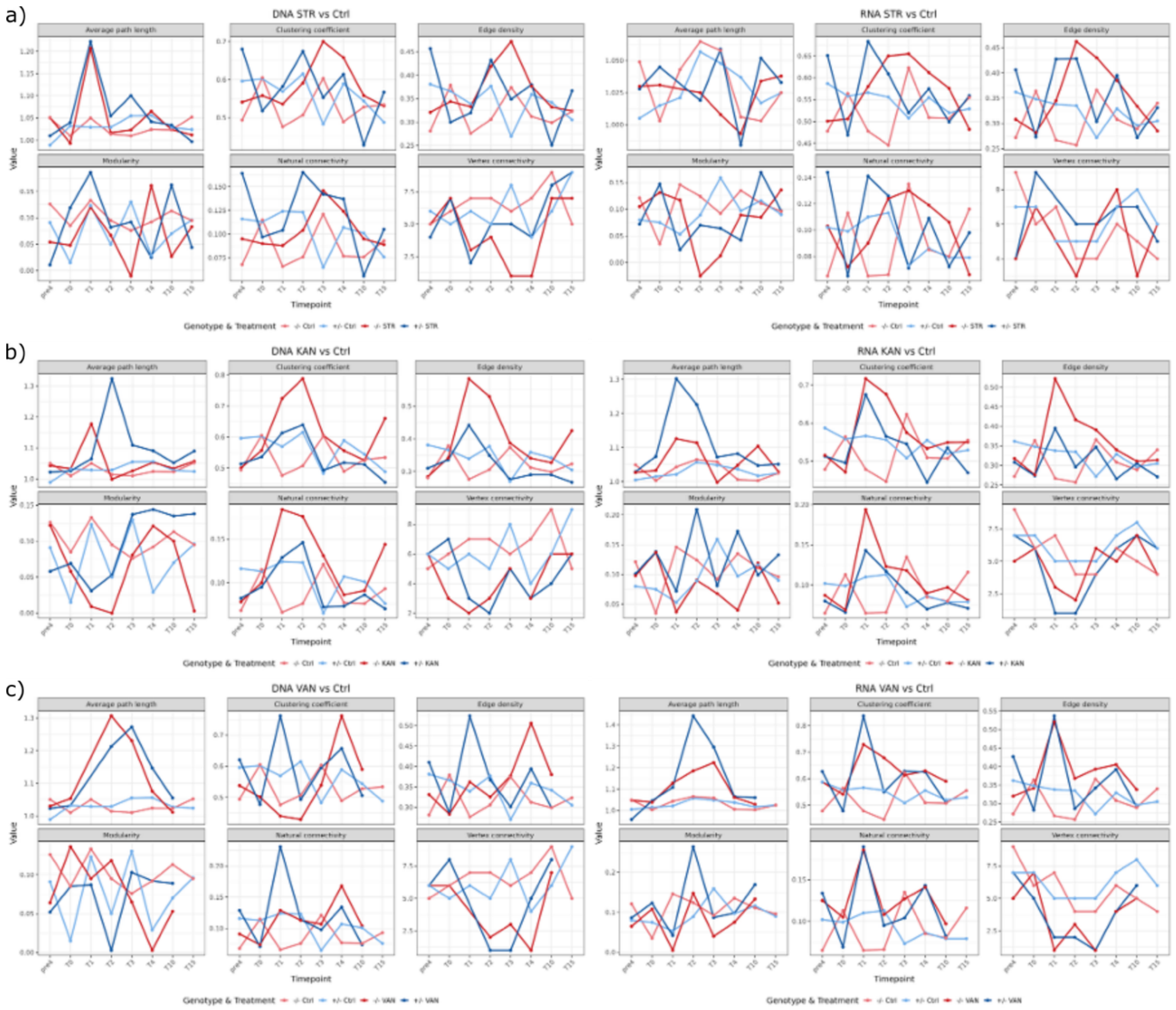

Supplementary Figure 19: Temporal dynamics of global network properties in 16S rRNA gene (DNA)- or transcript (RNA)-based microbial co-occurrence networks across all timepoints. Lines and points show genotype-specific networks, with colors indicating *B4galnt2*$^{-/-}$ vs *B4galnt2*$^{+/-}$ genotypes under treatment (dark red or blue) and control (light red or blue).

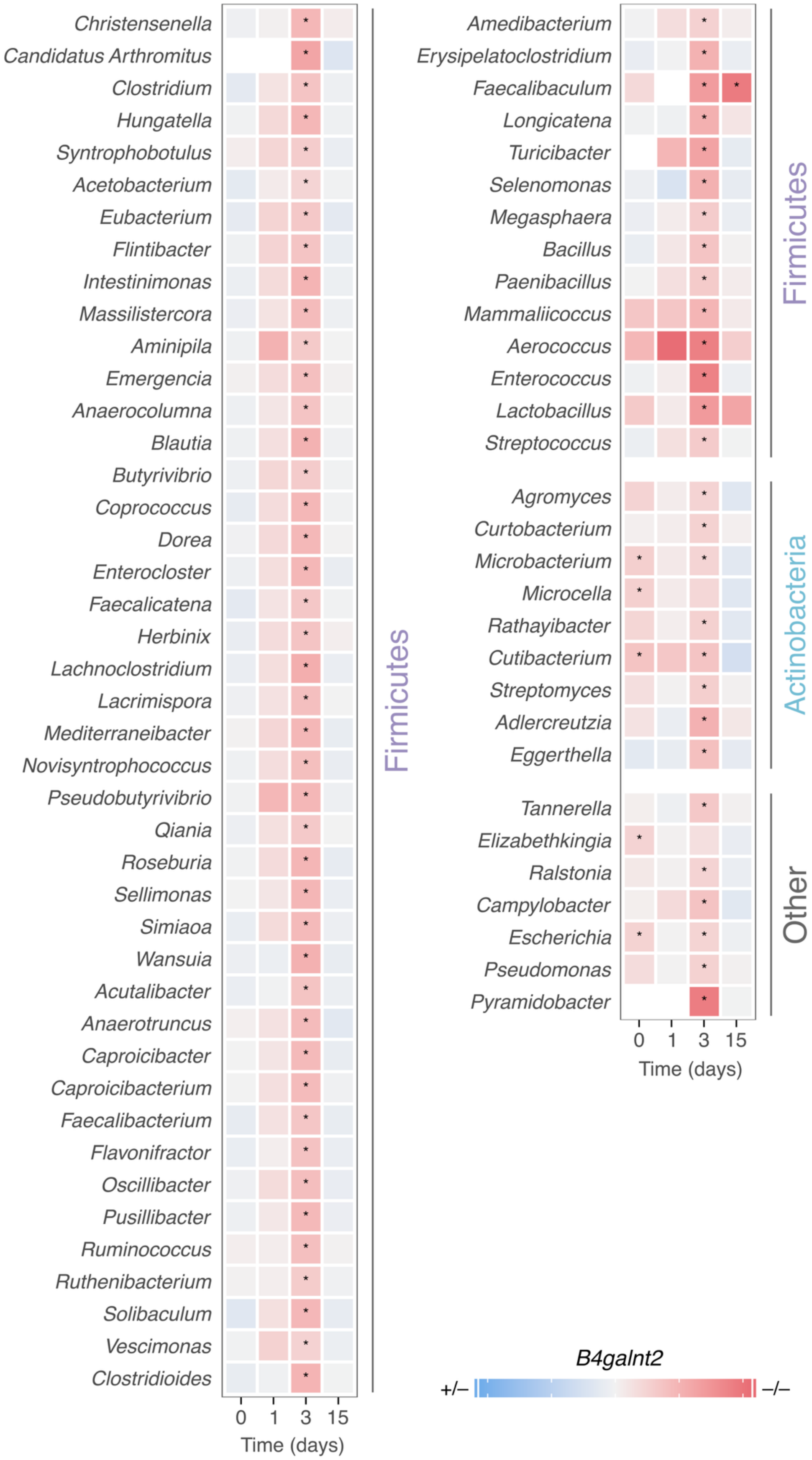

Supplementary Figure 20: Differently abundant genera between *B4galnt2* groups at different time points. Stars represent a $q_{val} < 0.25$.

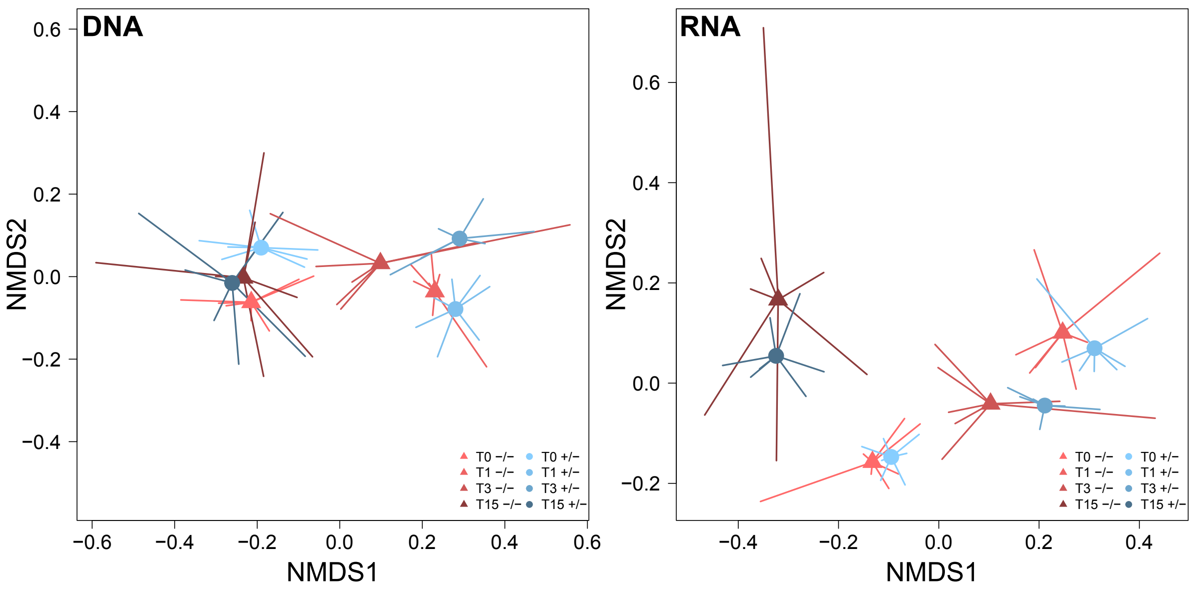

Supplementary Figure 21: Nonmetric multidimensional scaling (NMDS) plot of Bray–Curtis dissimilarity distances of CAZyme families

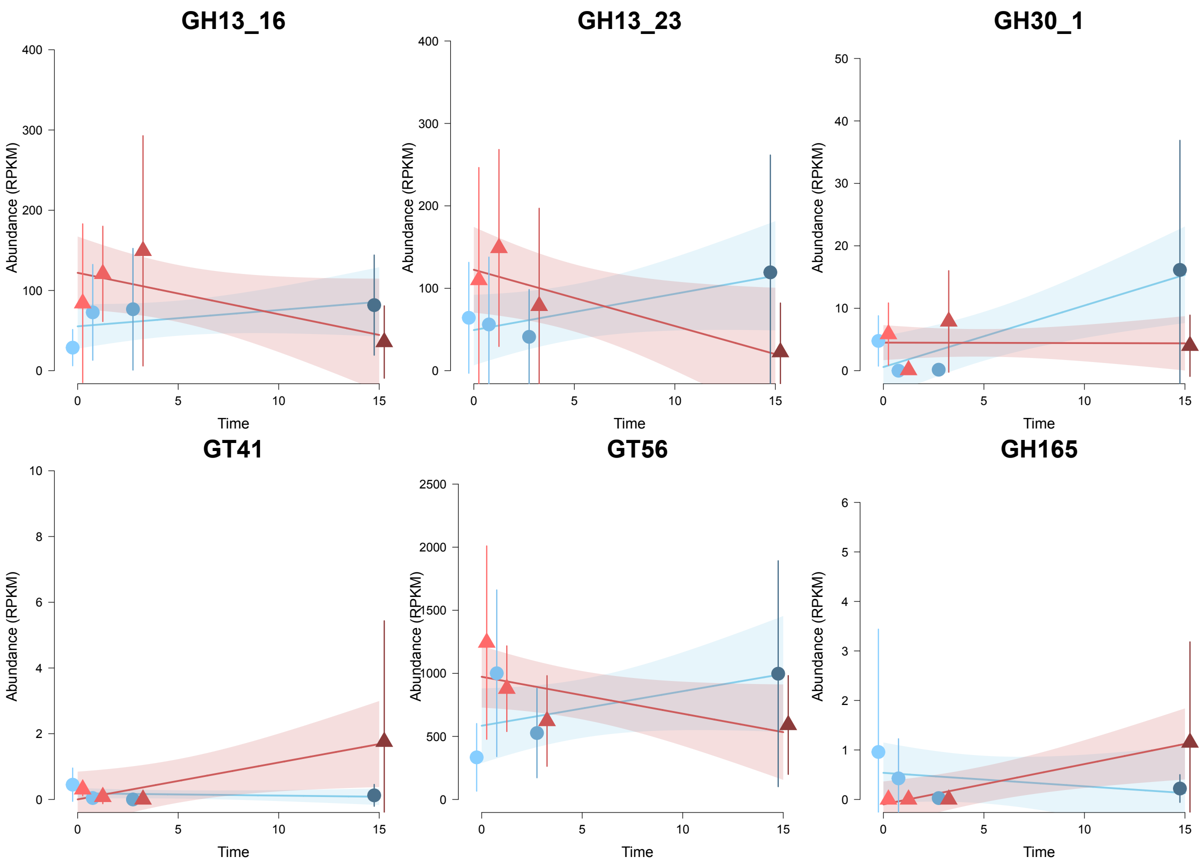

Supplementary Figure 22: Visualization of linear mixed-effect models showing changes in the significant CAZyme families at the metagenome level.

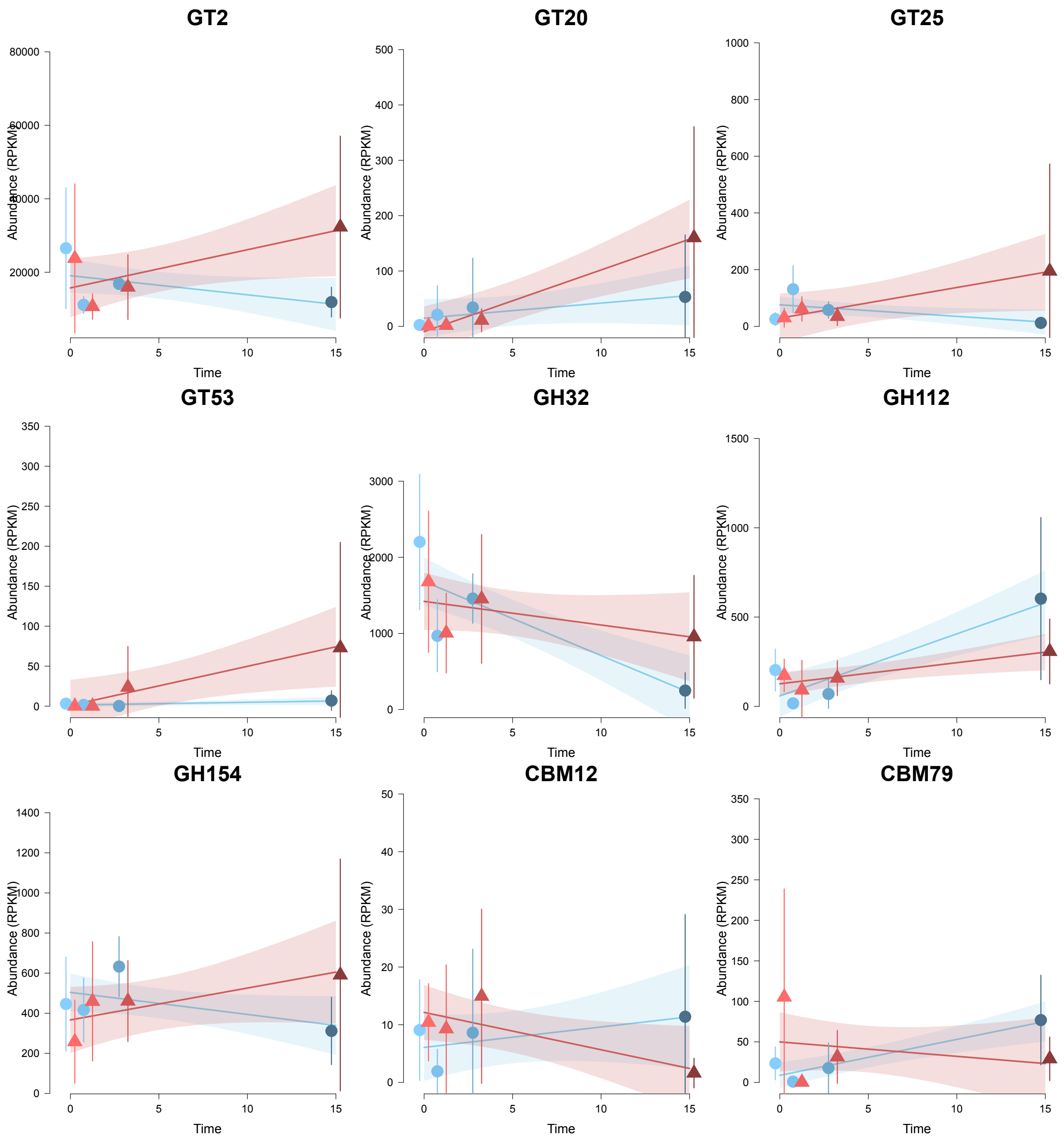

Supplementary Figure 23: Visualization of linear mixed-effect models showing changes in the significant CAZyme families at the metatranscriptome level.

Supplementary Table 1: Between-genotype comparisons of alpha diversity indices across time points

contrast	Time	estimate	SE	t.ratio	p.value	metric
(-/-) - (+/-)	0	-0.1429	7.0867	-0.0202	0.9842	observed
(-/-) - (+/-)	1	8.0000	7.0867	1.1289	0.2810	observed
(-/-) - (+/-)	3	-1.0000	7.0867	-0.1411	0.8901	observed
(-/-) - (+/-)	15	0.8571	7.0867	0.1210	0.9057	observed
(-/-) - (+/-)	0	-0.2139	0.3061	-0.6988	0.4980	diversity_shannon
(-/-) - (+/-)	1	0.0911	0.3061	0.2976	0.7711	diversity_shannon
(-/-) - (+/-)	3	0.7596	0.3061	2.4816	**0.0289**	diversity_shannon
(-/-) - (+/-)	15	0.1291	0.3061	0.4219	0.6806	diversity_shannon
(-/-) - (+/-)	0	-0.0160	0.0103	-1.5518	0.1467	evenness_simpson
(-/-) - (+/-)	1	-0.0028	0.0103	-0.2736	0.7890	evenness_simpson
(-/-) - (+/-)	3	0.0229	0.0103	2.2281	**0.0458**	evenness_simpson
(-/-) - (+/-)	15	0.0026	0.0103	0.2547	0.8032	evenness_simpson

Supplementary Table 2: Within-genotype temporal comparisons of alpha diversity indices

contrast	Genotype	estimate	SE	t.ratio	p.value	metric
1 - 0	-/-	-22.8571	7.1987	-3.1752	**0.0092**	observed
3 - 0	-/-	-10.4286	6.2344	-1.6728	0.2061	observed
15 - 0	-/-	-1.1429	5.8855	-0.1942	0.8471	observed
1 - 0	+/-	-31.0000	7.1987	-4.3063	**0.0004**	observed
3 - 0	+/-	-9.5714	6.2344	-1.5353	0.2669	observed
15 - 0	+/-	-2.1429	5.8855	-0.3641	0.7179	observed
1 - 0	-/-	-0.9035	0.2039	-4.4319	**0.0003**	diversity_shannon
3 - 0	-/-	-0.5111	0.2525	-2.0241	0.1009	diversity_shannon
15 - 0	-/-	0.1154	0.2601	0.4436	0.6600	diversity_shannon
1 - 0	+/-	-1.2086	0.2039	-5.9280	**0.0000**	diversity_shannon
3 - 0	+/-	-1.4846	0.2525	-5.8797	**0.0000**	diversity_shannon
15 - 0	+/-	-0.2277	0.2601	-0.8756	0.3871	diversity_shannon
1 - 0	-/-	-0.0173	0.0120	-1.4355	0.4793	evenness_simpson
3 - 0	-/-	-0.0011	0.0107	-0.1008	0.9203	evenness_simpson
15 - 0	-/-	0.0087	0.0093	0.9376	0.7094	evenness_simpson
1 - 0	+/-	-0.0304	0.0120	-2.5286	**0.0320**	evenness_simpson
3 - 0	+/-	-0.0400	0.0107	-3.7499	**0.0019**	evenness_simpson
15 - 0	+/-	-0.0099	0.0093	-1.0651	0.2939	evenness_simpson

Supplementary Table 3: Significantly differential KEGG orthology groups ($q_{val} < 0.25$) between *B4galnt2*$^{+/-}$ and *B4galnt2*$^{-/-}$ mice in metagenomics (MGX) and metatranscriptomics (MTX) samples:

feature	coef	stderr	qval	Time	Type	feature name
K03431	-2.4942	0.2628	0.0023	3	MGX	phosphoglucosamine_mutase_EC_5_4_2_10_
K02769	-3.1152	0.3858	0.0055	3	MGX	PTS_system_fructose_specific_IIB_component_EC_2_7_1_202_
K03763	-3.0113	0.3946	0.0062	3	MGX	DNA_polymerase_III_subunit_alpha_Gram_positive_type_EC_2_7_7_7_
K09698	-3.8020	0.5174	0.0066	3	MGX	nondiscriminating_glutamyl_tRNA_synthetase_EC_6_1_1_24_
K02795	-4.3719	0.6277	0.0087	3	MGX	PTS_system_mannose_specific_IIC_component
K00647	-5.7745	0.9853	0.0333	3	MGX	3_oxoacyl_acyl_carrier_protein_synthase_I_EC_2_3_1_41_
K01839	-4.8443	0.8725	0.0393	3	MGX	phosphopentomutase_EC_5_4_2_7_
K03086	-2.0384	0.3613	0.0393	3	MGX	RNA_polymerase_primary_sigma_factor
K11069	-3.2137	0.5870	0.0393	3	MGX	spermidine_putrescine_transport_system_substrate_binding_protein
K03486	-3.0216	0.5820	0.0545	3	MGX	GntR_family_transcriptional_regulator_trehalose_operon
K02075	-2.3877	0.4684	0.0554	3	MGX	zinc_manganese_transport_system_permease_protein
K02761	-3.1746	0.6269	0.0554	3	MGX	PTS_system_cellobiose_specific_IIC_component
K09762	-4.0797	0.8529	0.0799	3	MGX	hypothetical_protein
K07118	-2.0990	0.4739	0.1259	3	MGX	NO_NAME
K22393	-2.7612	0.6251	0.1259	3	MGX	NO_NAME
K03367	-2.7227	0.6254	0.1313	3	MGX	D_alanine_poly_phosphoribitol_ligase_subunit_1_EC_6_1_1_13_
K14188	-2.4713	0.5777	0.1402	3	MGX	D_alanine_poly_phosphoribitol_ligase_subunit_2_EC_6_1_1_13_
K15986	-1.7877	0.4390	0.1874	3	MGX	manganese_dependent_inorganic_pyrophosphatase_EC_3_6_1_1_
K01759	-2.7622	0.6935	0.1964	3	MGX	lactoylglutathione_lyase_EC_4_4_1_5_
K09158	2.0668	0.5178	0.1964	3	MGX	hypothetical_protein
K12555	-2.3813	0.6045	0.1981	3	MGX	penicillin_binding_protein_2A_EC_2_4_1_129_2_3_2_
K18891	-2.4978	0.6368	0.1981	3	MGX	ATP_binding_cassette_subfamily_B_multidrug_efflux_pump
K02770	-2.2754	0.5840	0.1981	3	MGX	PTS_system_fructose_specific_IIC_component
K02825	-2.5463	0.6664	0.2041	3	MGX	pyrimidine_operon_attenuation_protein_uracil_phosphoribosyltransferas
K03315	-2.6172	0.6873	0.2041	3	MGX	Na_H_antiporter_NhaC_family
K11068	-2.1543	0.5599	0.2041	3	MGX	hemolysin_III
K01609	1.3130	0.3557	0.2161	3	MGX	indole_3_glycerol_phosphate_synthase_EC_4_1_1_48_
K01916	-2.4652	0.6667	0.2161	3	MGX	NAD_synthase_EC_6_3_1_5_
K05340	-2.3759	0.6451	0.2161	3	MGX	glucose_uptake_protein
K06994	-2.3039	0.6210	0.2161	3	MGX	putative_drug_exporter_of_the_RND_superfamily
K11070	-1.9997	0.5443	0.2161	3	MGX	spermidine_putrescine_transport_system_permease_protein
K09787	-2.0535	0.5619	0.2165	3	MGX	hypothetical_protein
K04094	-1.7952	0.5015	0.2358	3	MGX	glucose_inhibited_division_protein_Gid
K01304	-2.0548	0.5772	0.2358	3	MGX	pyroglutamyl_peptidase
K03739	-2.1453	0.6035	0.2358	3	MGX	membrane_protein_involved_in_D_alanine_export
K07214	-2.5996	0.7366	0.2397	3	MGX	enterochelin_esterase_and_related_enzymes
K00244	-3.8552	0.5908	0.0662	3	MTX	fumarate_reductase_flavoprotein_subunit_EC_1_3_5_4_
K00364	-2.6945	0.5409	0.1283	3	MTX	GMP_reductase_EC_1_7_1_7_
K00615	-1.2315	0.3207	0.1477	3	MTX	transketolase_EC_2_2_1_1_
K00674	-1.7524	0.3783	0.1404	3	MTX	2_3_4_5_tetrahydropyridine_2_carboxylate_N_succinyltransferase_EC_2_3

feature	coef	stderr	qval	Time	Type	feature name
K00721	0.9637	0.2626	0.1477	3	MTX	dolichol_phosphate_mannosyltransferase_EC_2_4_1_83_
K00868	-1.3280	0.3607	0.1477	3	MTX	pyridoxine_kinase_EC_2_7_1_35_
K00965	-1.2051	0.3164	0.1477	3	MTX	UDPglucose_hexose_1_phosphate_uridylyltransferase_EC_2_7_7_12_
K01512	-1.6350	0.4027	0.1457	3	MTX	acylphosphatase_EC_3_6_1_7_
K01779	-1.5107	0.3016	0.1283	3	MTX	aspartate_racemase_EC_5_1_1_13_
K01784	-1.1491	0.3134	0.1477	3	MTX	UDP_glucose_4_epimerase_EC_5_1_3_2_
K02236	-1.3120	0.3264	0.1477	3	MTX	leader_peptidase_prepilin_peptidase_N_methyltransferase
K02242	-1.1993	0.2391	0.1283	3	MTX	competence_protein_ComFC
K02503	-0.7729	0.2129	0.1477	3	MTX	Hit_like_protein_involved_in_cell_cycle_regulation
K02755	-1.4971	0.3673	0.1457	3	MTX	PTS_system_beta_glucoside_specific_IIA_component_EC_2_7_1_
K02756	-1.4971	0.3673	0.1457	3	MTX	PTS_system_beta_glucoside_specific_IIB_component_EC_2_7_1_
K02757	-1.4971	0.3673	0.1457	3	MTX	PTS_system_beta_glucoside_specific_IIC_component
K02761	-2.5413	0.5838	0.1457	3	MTX	PTS_system_cellobiose_specific_IIC_component
K02769	-2.0346	0.5606	0.1477	3	MTX	PTS_system_fructose_specific_IIB_component_EC_2_7_1_202_
K02793	-1.5523	0.3489	0.1457	3	MTX	PTS_system_mannose_specific_IIA_component_EC_2_7_1_191_
K03216	-1.1881	0.3320	0.1477	3	MTX	RNA_methyltransferase_TrmH_family_group_2
K03320	0.6942	0.1663	0.1457	3	MTX	ammonium_transporter_Amt_family
K03321	1.0714	0.2807	0.1477	3	MTX	sulfate_permease_SulP_family
K03571	-1.3223	0.3683	0.1477	3	MTX	rod_shape_determining_protein_MreD
K03592	-2.5427	0.6926	0.1477	3	MTX	PmbA_protein
K03710	-1.5096	0.4093	0.1477	3	MTX	GntR_family_transcriptional_regulator
K03722	-1.3676	0.3439	0.1477	3	MTX	ATP_dependent_DNA_helicase_DinG
K03763	-2.2536	0.5359	0.1457	3	MTX	DNA_polymerase_III_subunit_alpha_Gram_positive_type_EC_2_7_7_7_
K03817	-1.4297	0.3461	0.1457	3	MTX	ribosomal_protein_serine_acetyltransferase
K04042	-1.5510	0.4159	0.1477	3	MTX	bifunctional_UDP_N_acetylglucosamine_pyrophosphorylase_Glucosamine
K04094	-1.9369	0.5340	0.1477	3	MTX	glucose_inhibited_division_protein_Gid
K06148	-1.3344	0.3728	0.1477	3	MTX	ATP_binding_cassette_subfamily_C_bacterial
K06183	-1.4424	0.3835	0.1477	3	MTX	ribosomal_small_subunit_pseudouridine_synthase_A
K06861	0.8149	0.2197	0.1477	3	MTX	lipopolysaccharide_export_system_ATP_binding_protein_EC_3_6_3_
K07078	-1.7429	0.3123	0.1276	3	MTX	NO_NAME
K07090	-2.5573	0.6023	0.1457	3	MTX	NO_NAME
K07473	-4.5736	0.9564	0.1404	3	MTX	DNA_damage_inducible_protein_J
K08602	-1.6880	0.3934	0.1457	3	MTX	oligoendopeptidase_F
K09773	-1.3738	0.3531	0.1477	3	MTX	hypothetical_protein
K09963	-1.0802	0.2923	0.1477	3	MTX	hypothetical_protein
K10026	1.6134	0.4382	0.1477	3	MTX	7_carboxy_7_deazaguanine_synthase_EC_4_3_99_3_
K11069	-2.4649	0.5727	0.1457	3	MTX	spermidine_putrescine_transport_system_substrate_binding_protein
K13694	1.2711	0.2725	0.1404	3	MTX	NO_NAME
K14188	-1.5012	0.4191	0.1477	3	MTX	D_alanine_poly_phosphoribitol_ligase_subunit_2_EC_6_1_1_13_
K15986	-1.4924	0.3994	0.1477	3	MTX	manganese_dependent_inorganic_pyrophosphatase_EC_3_6_1_1_
K18330	1.8784	0.5229	0.1477	3	MTX	NO_NAME
K02077	-1.3940	0.3909	0.1477	3	MTX	zinc_manganese_transport_system_substrate_binding_protein

feature	coef	stderr	qval	Time	Type	feature name
K03601	-0.6459	0.1815	0.1477	3	MTX	exodeoxyribonuclease_VII_large_subunit_EC_3_1_11_6_
K00282	1.3189	0.3726	0.1492	3	MTX	glycine_dehydrogenase_subunit_1_EC_1_4_4_2_
K01488	-1.6203	0.4629	0.1568	3	MTX	adenosine_deaminase_EC_3_5_4_4_
K01304	-1.1414	0.3337	0.1734	3	MTX	pyroglutamyl_peptidase
K02244	-1.0339	0.3020	0.1734	3	MTX	competence_protein_ComGB
K00383	-1.1749	0.3481	0.1762	3	MTX	glutathione_reductase_NADPH_EC_1_8_1_7_
K00963	-1.6151	0.4760	0.1762	3	MTX	UTP_glucose_1_phosphate_uridylyltransferase_EC_2_7_7_9_
K12268	-1.2715	0.3761	0.1762	3	MTX	accessory_secretory_protein_Asp1
K16169	-1.2803	0.3800	0.1762	3	MTX	NO_NAME
K02551	0.9121	0.2721	0.1784	3	MTX	2_succinyl_5_enolpyruvyl_6_hydroxy_3_cyclohexene_1_carboxylate_synth
K07335	-1.4943	0.4524	0.1910	3	MTX	basic_membrane_protein_A_and_related_proteins
K02031	-2.1381	0.6551	0.1975	3	MTX	peptide_nickel_transport_system_ATP_binding_protein
K04758	-2.1409	0.6566	0.1975	3	MTX	ferrous_iron_transport_protein_A
K01200	0.8156	0.2544	0.1975	3	MTX	pullulanase
K01759	-2.2767	0.7138	0.1975	3	MTX	lactoylglutathione_lyase_EC_4_4_1_5_
K02075	-1.3647	0.4290	0.1975	3	MTX	zinc_manganese_transport_system_permease_protein
K02433	-0.8169	0.2571	0.1975	3	MTX	aspartyl_tRNA_Asn_glutamyl_tRNA_Gln_amidotransferase_subunit_A_EC_6
K02795	-1.5616	0.4882	0.1975	3	MTX	PTS_system_mannose_specific_IIC_component
K02796	-1.2295	0.3804	0.1975	3	MTX	PTS_system_mannose_specific_IID_component
K05823	-1.2038	0.3785	0.1975	3	MTX	N_acetyldiaminopimelate_deacetylase_EC_3_5_1_47_
K07154	1.7253	0.5390	0.1975	3	MTX	NO_NAME
K09762	-1.9787	0.6130	0.1975	3	MTX	hypothetical_protein
K17076	-1.3216	0.4140	0.1975	3	MTX	putative_lysine_transport_system_ATP_binding_protein_EC_3_6_3_
K07738	-1.1818	0.3747	0.2028	3	MTX	transcriptional_repressor_NrdR
K20811	-1.2911	0.4110	0.2043	3	MTX	NO_NAME
K00158	-1.5301	0.4896	0.2043	3	MTX	pyruvate_oxidase_EC_1_2_3_3_
K00974	-2.1043	0.6781	0.2046	3	MTX	tRNA_nucleotidyltransferase_CCA_adding_enzyme_EC_2_7_7_72_3_1_3_3_
K01595	-1.5121	0.4833	0.2043	3	MTX	phosphoenolpyruvate_carboxylase_EC_4_1_1_31_
K02034	-2.1935	0.7033	0.2043	3	MTX	peptide_nickel_transport_system_permease_protein
K02246	-1.5162	0.4890	0.2046	3	MTX	competence_protein_ComGD
K03892	-0.8731	0.2820	0.2046	3	MTX	ArsR_family_transcriptional_regulator
K03574	-1.2219	0.3963	0.2063	3	MTX	7_8_dihydro_8_oxoguanine_triphosphatase
K01195	0.9251	0.3010	0.2073	3	MTX	beta_glucuronidase_EC_3_2_1_31_
K00638	1.8086	0.5950	0.2156	3	MTX	chloramphenicol_O_acetyltransferase
K05946	-1.5338	0.5060	0.2156	3	MTX	N_acetylglucosaminyldiphosphoundecaprenol
K08996	-1.6545	0.5452	0.2156	3	MTX	putative_membrane_protein
K02245	-0.8255	0.2746	0.2200	3	MTX	competence_protein_ComGC
K07533	-1.2106	0.4024	0.2200	3	MTX	foldase_protein_PrsA
K16012	-1.1279	0.3789	0.2291	3	MTX	ATP_binding_cassette_subfamily_C_bacterial_CydC
K02240	-1.2252	0.4125	0.2291	3	MTX	competence_protein_ComFA
K08591	-1.1604	0.3925	0.2294	3	MTX	glycerol_3_phosphate_acyltransferase_PlsY_EC_2_3_1_15_
K09702	0.9898	0.3342	0.2294	3	MTX	hypothetical_protein

feature	coef	stderr	qval	Time	Type	feature name
K11645	2.1842	0.7417	0.2308	3	MTX	fructose_bisphosphate_aldolase_class_I_EC_4_1_2_13_
K01006	-1.6979	0.5778	0.2308	3	MTX	pyruvate_orthophosphate_dikinase_EC_2_7_9_1_
K01607	-1.7366	0.5930	0.2308	3	MTX	4_carboxymuconolactone_decarboxylase_EC_4_1_1_44_
K06143	1.4202	0.4846	0.2308	3	MTX	inner_membrane_protein
K06518	1.0686	0.3669	0.2352	3	MTX	holin_like_protein
K02839	1.4811	0.5122	0.2388	3	MTX	peptide_chain_release_factor_RF_H
K12308	-1.3623	0.4709	0.2388	3	MTX	beta_galactosidase_EC_3_2_1_23_
K01639	0.7375	0.2570	0.2458	3	MTX	N_acetylneuraminate_lyase_EC_4_1_3_3_
K00873	-3.0190	0.5016	0.0668	15	MTX	pyruvate_kinase_EC_2_7_1_40_
K14441	-5.4877	0.9641	0.0668	15	MTX	NO_NAME
K03671	-2.3314	0.4874	0.1409	15	MTX	thioredoxin_1
K03705	-2.6486	0.5564	0.1409	15	MTX	heat_inducible_transcriptional_repressor
K05808	-1.8390	0.4253	0.2250	15	MTX	putative_sigma_54_modulation_protein
K10118	-5.6023	1.3246	0.2250	15	MTX	raffinose_stachyose_melibiose_transport_system_permease_protein

Supplementary Table 4: Genotype-specific temporal changes in CAZyme family abundance and expression based on metagenomic and metatranscriptomic analyses

Data	CAZyme	Model Factors	DenDF	F	P	R2	adj.R2
DNA	GT56	Genotype	22.45	3.2983	0.0827	0.0904	0.2015
		Time	40.00	0.0063	0.9373		
		Genotype:Time	40.00	5.5412	0.0236		
DNA	GH13_23	Genotype	52.00	4.5372	0.0379	0.1145	0.1145
		Time	52.00	0.2949	0.5894		
		Genotype:Time	52.00	6.2722	0.0154		
DNA	GH30_1	Genotype	27.56	1.7563	0.1960	0.1968	0.2068
		Time	40.00	6.6589	0.0136		
		Genotype:Time	40.00	6.8645	0.0124		
DNA	GH13_16	Genotype	52.00	6.0528	0.0172	0.1166	0.1166
		Time	52.00	0.7817	0.3807		
		Genotype:Time	52.00	4.1413	0.0470		
DNA	GT41	Genotype	25.32	0.1648	0.6881	0.1394	0.1859
		Time	40.00	3.5586	0.0665		
		Genotype:Time	40.00	4.5702	0.0387		
DNA	GH165	Genotype	22.60	2.2304	0.1492	0.0944	0.2015
		Time	40.00	1.2617	0.2680		
		Genotype:Time	40.00	5.0668	0.0300		
RNA	GH32	Genotype	52.00	1.0828	0.3029	0.2621	0.2621
		Time	52.00	15.3646	0.0003		
		Genotype:Time	52.00	4.0969	0.0481		
RNA	GT2	Genotype	25.58	0.4642	0.5018	0.1311	0.1734
		Time	40.00	0.7398	0.3948		
		Genotype:Time	40.00	6.7191	0.0133		
RNA	GH112	Genotype	52.00	0.9917	0.3239	0.3835	0.3835
		Time	52.00	27.0588	0.0000		
		Genotype:Time	52.00	6.5813	0.0132		
RNA	GH154	Genotype	28.12	1.9976	0.1685	0.0785	0.0815
		Time	40.00	0.1496	0.7010		
		Genotype:Time	40.00	4.5345	0.0394		
RNA	GT25	Genotype	52.00	1.0081	0.3200	0.1208	0.1208
		Time	52.00	1.2126	0.2759		
		Genotype:Time	52.00	5.9273	0.0184		
RNA	CBM79	Genotype	27.54	4.0283	0.0547	0.1131	0.1244
		Time	40.00	1.0134	0.3201		
		Genotype:Time	40.00	5.5462	0.0235		
RNA	CBM12	Genotype	52.00	2.5330	0.1175	0.0771	0.0771
		Time	52.00	0.3480	0.5578		
		Genotype:Time	52.00	4.0525	0.0493		
RNA	GT20	Genotype	23.15	0.5867	0.4515	0.2487	0.3266

Data	CAZyme	Model Factors	DenDF	F	P	R2	adj.R2
		Time	40.00	14.4728	0.0005		
		Genotype:Time	40.00	5.3249	0.0263		
RNA	GT53	Genotype	52.00	0.0005	0.9822	0.1920	0.1920
		Time	52.00	5.9251	0.0184		
		Genotype:Time	52.00	4.4575	0.0396		

Supplementary Table 5: Between-genotype comparisons of individual CAZyme families at single time points

Data	Time	#	CAZyme	W	P	PFDR	Mean_neg	Mean_pos
DNA	T0	1	AA1	43	0.0175	0.3706	884.0120	376.0066
DNA	T0	4	GH13_19	45	0.0070	0.3177	908.9164	261.6651
DNA	T0	5	GH13_21	45	0.0070	0.3177	664.8179	167.0853
DNA	T0	13	GH15	46	0.0041	0.3177	142.7514	33.7098
DNA	T0	14	GH153	43	0.0175	0.3706	1152.1471	377.8975
DNA	T0	33	GH37	45	0.0070	0.3177	880.1325	399.7845
DNA	T0	34	GH38	49	0.0006	0.1853	1045.2512	377.0980
DNA	T0	35	GH4	47	0.0023	0.3177	2674.8899	1046.1567
DNA	T0	42	AA3	44	0.0111	0.3201	155.0126	39.4960
DNA	T0	59	GH63	44	0.0111	0.3201	759.7016	383.1110
DNA	T0	64	GH77	42	0.0262	0.4633	1104.3999	572.0390
DNA	T0	79	GT20	43	0.0175	0.3706	818.8680	283.5264
DNA	T0	80	GT26	44	0.0111	0.3201	1012.7226	362.0395
DNA	T0	88	GT51	41	0.0379	0.5736	5857.7865	2557.2857
DNA	T0	89	GT56	43	0.0175	0.3706	1243.6257	333.8149
DNA	T0	109	CE4	42	0.0262	0.4633	2644.6139	1833.0638
DNA	T0	145	GH137	38.5	0.0307	0.5142	0.6102	0.0000
DNA	T0	212	GH30_8	41	0.0379	0.5736	13.5899	3.7623
DNA	T0	222	GH43_9	44	0.0111	0.3201	17.9114	3.3332
DNA	T0	237	GH93	45	0.0070	0.3177	5.3540	0.8622
DNA	T0	305	GH156	6	0.0187	0.3709	0.1526	0.9953
DNA	T1	23	GH26	7	0.0262	0.8003	891.0369	1413.6272
DNA	T1	104	CBM67	7	0.0262	0.8003	1733.1574	2646.4615
DNA	T1	160	CBM2	42	0.0262	0.8003	84.1786	38.3208
DNA	T1	209	GH30_8	42	0.0114	0.8003	7.4700	0.0000
DNA	T1	215	GH43_37	42.5	0.0199	0.8003	3.5424	0.5927
DNA	T1	217	GH43_9	42.5	0.0199	0.8003	10.0829	0.6417
DNA	T1	227	GH93	38.5	0.0307	0.8003	7.1365	0.0000
DNA	T1	242	GT73	42	0.0114	0.8003	2.3008	0.0000
DNA	T3	4	GH13_19	41	0.0379	0.3390	1038.4818	392.0541
DNA	T3	6	GH13_29	41	0.0379	0.3390	286.1139	52.9065
DNA	T3	15	GH158	6	0.0175	0.3390	115.1205	255.2888
DNA	T3	19	GH20	8	0.0379	0.3390	1711.8689	2660.3708
DNA	T3	38	GH43_10	7	0.0262	0.3390	1203.9532	2490.9571
DNA	T3	44	GH43_29	7	0.0262	0.3390	1207.9073	2492.3042
DNA	T3	67	GH88	8	0.0379	0.3390	1474.8320	2965.4859
DNA	T3	72	GH95	5	0.0111	0.3390	786.3407	1408.9192
DNA	T3	73	GH97	6	0.0175	0.3390	4955.1099	10286.4439
DNA	T3	78	GT2	6	0.0175	0.3390	16207.4474	19683.0165

Data	Time	#	CAZyme	W	P	PFDR	Mean_neg	Mean_pos
DNA	T3	82	GT3	4	0.0070	0.3390	340.0655	604.7103
DNA	T3	102	CBM6	7	0.0262	0.3390	1630.8245	3448.3201
DNA	T3	116	GH10	7	0.0262	0.3390	3453.2157	9805.3406
DNA	T3	172	GT32	8	0.0379	0.3390	1200.0553	2047.0521
DNA	T3	175	PL10_1	8	0.0379	0.3390	316.8318	749.2935
DNA	T3	177	PL12_2	6	0.0175	0.3390	262.7161	687.1152
DNA	T3	180	PL15_2	8	0.0379	0.3390	78.8684	172.8893
DNA	T3	190	GH13_36	41	0.0407	0.3390	19.7015	3.8226
DNA	T3	193	GH13_41	40	0.0407	0.3390	13.5923	0.0569
DNA	T3	198	GH143	8	0.0379	0.3390	9.1945	17.4012
DNA	T3	201	CBM23	45	0.0090	0.3390	16.6920	0.3248
DNA	T3	208	GH30_1	43.5	0.0139	0.3390	7.8881	0.1603
DNA	T3	213	GH39	42	0.0262	0.3390	22.4345	3.0942
DNA	T3	226	CBM36	42.5	0.0199	0.3390	29.9595	0.4007
DNA	T3	227	GH5_37	41	0.0400	0.3390	13.0778	2.0847
DNA	T3	235	GH93	44	0.0095	0.3390	17.1626	0.1786
DNA	T3	239	GT10	43	0.0175	0.3390	38.6084	13.2089
DNA	T3	247	GT27	7	0.0262	0.3390	23.8761	97.1858
DNA	T3	255	CBM51	42	0.0262	0.3390	78.3387	34.5042
DNA	T3	260	PL12_1	45	0.0070	0.3390	25.5788	1.0432
DNA	T3	269	PL9	42.5	0.0238	0.3390	7.1724	0.2487
DNA	T3	270	PL9_1	41	0.0379	0.3390	4.2136	1.2368
DNA	T3	276	CE14	44	0.0147	0.3390	14.0935	0.5351
DNA	T3	280	GH11	42.5	0.0199	0.3390	24.1669	0.7190
DNA	T3	282	GH113	43	0.0187	0.3390	12.6461	0.2607
DNA	T3	292	PL10	6	0.0175	0.3390	32.5583	87.0146
DNA	T15	67	GH88	41	0.0379	0.9907	296.8736	137.4712
DNA	T15	132	PL4	42	0.0263	0.9907	64.3719	5.2025
DNA	T15	167	GH5_13	43	0.0209	0.9907	15.0550	1.0018
DNA	T15	180	PL15_2	46	0.0067	0.9907	13.3835	1.5062
DNA	T15	198	GH143	44	0.0151	0.9907	3.5809	0.4630
DNA	T15	264	PL1_8	5	0.0151	0.9907	0.3777	1.5277
RNA	T0	27	GH33	6	0.0175	0.4757	470.8959	1036.5778
RNA	T0	69	GT11	2	0.0023	0.4351	172.4054	347.1638
RNA	T0	70	GT111	6	0.0175	0.4757	140.7895	257.9678
RNA	T0	98	CBM62	6	0.0175	0.4757	975.4290	2979.5233
RNA	T0	104	CE4	6	0.0175	0.4757	1107.5988	1531.8976
RNA	T0	117	GH13	8	0.0379	0.4757	27164.4361	38959.7800
RNA	T0	119	GH133	8	0.0379	0.4757	273.5316	438.9086
RNA	T0	124	GH13_42	42	0.0262	0.4757	31.1851	10.8976
RNA	T0	144	GH111	9	0.0500	0.4757	0.5110	1.8691
RNA	T0	154	GH13_6	41	0.0379	0.4757	23.8827	10.0979
RNA	T0	156	GH16	6	0.0175	0.4757	374.2443	686.4773
RNA	T0	157	GH161	8	0.0379	0.4757	7.1672	14.9135
RNA	T0	188	PL29	3	0.0041	0.4351	71.5338	289.9333
RNA	T0	210	GH17	7	0.0262	0.4757	2.1607	7.3697
RNA	T0	217	CBM27	8	0.0407	0.4757	7.5765	30.9732
RNA	T0	222	GH43_33	8	0.0379	0.4757	5.1596	9.6652
RNA	T0	224	GH43_9	42	0.0262	0.4757	16.8194	3.4937
RNA	T0	228	GH55	3	0.0041	0.4351	2.3085	7.1188
RNA	T0	257	GT73	4	0.0070	0.4757	6.5326	19.4962
RNA	T0	259	GT87	7	0.0291	0.4757	0.7017	19.3105

Data	Time	#	CAZyme	W	P	PFDR	Mean_neg	Mean_pos
RNA	T0	265	PL12_3	43	0.0175	0.4757	5.5148	1.6048
RNA	T0	270	PL37	40	0.0407	0.4757	4.6445	0.5957
RNA	T0	276	CBM68	41	0.0387	0.4757	6.4850	0.3415
RNA	T0	277	CBM74	41	0.0379	0.4757	12.8996	4.1347
RNA	T0	286	GH101	5	0.0111	0.4757	11.9492	110.0822
RNA	T0	300	GH156	8	0.0407	0.4757	0.9513	2.7700
RNA	T1	46	CBM34	41	0.0379	0.6550	37.9698	18.3468
RNA	T1	101	AA7	38.5	0.0307	0.6550	8.0559	0.0000
RNA	T1	143	GH110	6	0.0175	0.6550	178.5305	339.1168
RNA	T1	146	GH120	6	0.0175	0.6550	165.4903	619.0257
RNA	T1	148	GH136	8	0.0379	0.6550	54.8872	121.9159
RNA	T1	164	GH30_6	8	0.0379	0.6550	13.2998	41.4137
RNA	T1	207	GH19	38.5	0.0307	0.6550	9.2200	0.0000
RNA	T1	212	GH30_8	45	0.0064	0.6550	6.3712	0.0111
RNA	T1	217	GH43_33	7	0.0262	0.6550	29.0138	86.3045
RNA	T1	219	GH43_9	41	0.0365	0.6550	5.9014	0.6150
RNA	T1	236	GH98	42	0.0263	0.6550	15.7033	0.8912
RNA	T1	241	GT23	41.5	0.0330	0.6550	5.2190	1.0814
RNA	T1	250	CBM51	4	0.0070	0.6550	113.4684	325.6578
RNA	T1	261	PL9	40.5	0.0452	0.6550	1.3010	0.1511
RNA	T1	269	CE14	38.5	0.0307	0.6550	0.6753	0.0000
RNA	T1	279	GH129	42	0.0114	0.6550	0.0973	0.0000
RNA	T1	284	GH156	6	0.0175	0.6550	20.2760	71.1823
RNA	T3	13	GH16_3	7	0.0262	0.3390	506.9678	914.7304
RNA	T3	16	GH20	2	0.0023	0.3390	2723.1714	4955.1277
RNA	T3	20	GH28	6	0.0175	0.3390	3221.5366	5625.8494
RNA	T3	40	GH43_29	8	0.0379	0.3390	1343.1197	1949.7440
RNA	T3	42	GH43_31	6	0.0175	0.3390	992.6047	1716.8572
RNA	T3	62	GH88	7	0.0262	0.3390	2241.7429	3585.3303
RNA	T3	67	GH95	5	0.0111	0.3390	1600.6613	2410.9947
RNA	T3	68	GH97	5	0.0111	0.3390	8410.0816	13701.4156
RNA	T3	74	GT3	6	0.0175	0.3390	558.8933	820.7108
RNA	T3	77	GT4	8	0.0379	0.3390	6985.6546	9850.9613
RNA	T3	91	PL6	8	0.0379	0.3390	346.1552	1331.6306
RNA	T3	101	AA7	45	0.0070	0.3390	63.4373	1.6522
RNA	T3	106	CE7	5	0.0111	0.3390	1308.6618	2492.5642
RNA	T3	112	GH108	4	0.0070	0.3390	366.3385	971.2938
RNA	T3	148	GH138	7	0.0262	0.3390	67.0431	148.4125
RNA	T3	149	GH13_18	5	0.0111	0.3390	118.5151	419.9550
RNA	T3	157	GH163	6	0.0175	0.3390	41.3943	103.9877
RNA	T3	164	GH37	6	0.0175	0.3390	110.5853	287.7382
RNA	T3	180	PL10	8	0.0379	0.3390	23.3038	46.7345
RNA	T3	183	PL12_2	6	0.0175	0.3390	193.6464	492.3828
RNA	T3	186	PL15_2	8	0.0379	0.3390	129.1336	315.7993
RNA	T3	189	PL38	8	0.0379	0.3390	67.6323	134.6331
RNA	T3	192	CBM9	41	0.0400	0.3390	14.1182	1.3914
RNA	T3	194	GH13_41	45	0.0098	0.3390	5.4841	0.3154
RNA	T3	203	CBM23	41	0.0379	0.3390	10.6748	2.3142
RNA	T3	213	GH30_5	40	0.0338	0.3390	0.1967	0.0080
RNA	T3	216	GH39	41	0.0379	0.3390	33.0493	9.8301
RNA	T3	221	GH43_37	8	0.0379	0.3390	5.8132	13.6146
RNA	T3	239	GH93	44	0.0130	0.3390	12.9062	1.7534

Data	Time	#	CAZyme	W	P	PFDR	Mean_neg	Mean_pos
RNA	T3	240	CBM41	41	0.0365	0.3390	40.4079	3.5669
RNA	T3	248	GT27	7	0.0262	0.3390	15.1893	59.8762
RNA	T3	265	PL27	4	0.0070	0.3390	7.9512	33.4700
RNA	T15	10	GH144	7	0.0262	0.9301	319.5889	845.4960
RNA	T15	19	GH26	42	0.0262	0.9301	444.9124	167.2353
RNA	T15	27	GH33	43	0.0175	0.9301	782.9647	170.4048
RNA	T15	30	GH38	6	0.0175	0.9301	171.2936	694.7440
RNA	T15	35	GH43_10	43	0.0175	0.9301	1708.0342	421.9902
RNA	T15	72	GT2	46	0.0041	0.6690	32313.1861	11885.4689
RNA	T15	113	GH109	46	0.0041	0.6690	89.8219	11.1890
RNA	T15	127	GH25	7	0.0262	0.9301	271.7867	359.9120
RNA	T15	175	GT10	7	0.0291	0.9301	0.9740	3.6242
RNA	T15	222	GH43_37	43	0.0213	0.9301	4.8995	1.1413
RNA	T15	296	GH156	9	0.0407	0.9301	0.0894	0.2814

Supplementary Table 6: Sample metadata

Sample_ID	Group	Genotype	Sex	Mouse_ID
B4galnt2_ABX1_KAN_M15	KAN	-/-	m	M15
B4galnt2_ABX1_KAN_M16	KAN	-/-	f	M16
B4galnt2_ABX1_KAN_M17	KAN	-/-	f	M17
B4galnt2_ABX1_KAN_M18	KAN	-/-	m	M18
B4galnt2_ABX1_KAN_M19	KAN	-/-	f	M19
B4galnt2_ABX1_KAN_M20	KAN	-/-	m	M20
B4galnt2_ABX1_KAN_M21	KAN	-/-	f	M21
B4galnt2_ABX1_KAN_M22	KAN	+/-	f	M22
B4galnt2_ABX1_KAN_M23	KAN	+/-	m	M23
B4galnt2_ABX1_KAN_M24	KAN	+/-	f	M24
B4galnt2_ABX1_KAN_M25	KAN	+/-	m	M25
B4galnt2_ABX1_KAN_M26	KAN	+/-	m	M26
B4galnt2_ABX1_KAN_M27	KAN	+/-	f	M27
B4galnt2_ABX1_KAN_M28	KAN	+/-	m	M28
B4galnt2_ABX1_Ctrl_M29	Ctrl	-/-	m	M29
B4galnt2_ABX1_Ctrl_M30	Ctrl	-/-	m	M30
B4galnt2_ABX1_Ctrl_M31	Ctrl	-/-	f	M31
B4galnt2_ABX1_Ctrl_M32	Ctrl	-/-	m	M32
B4galnt2_ABX1_Ctrl_M33	Ctrl	-/-	m	M33
B4galnt2_ABX1_Ctrl_M34	Ctrl	-/-	f	M34
B4galnt2_ABX1_Ctrl_M35	Ctrl	-/-	f	M35
B4galnt2_ABX1_Ctrl_M36	Ctrl	+/-	m	M36
B4galnt2_ABX1_Ctrl_M37	Ctrl	+/-	f	M37
B4galnt2_ABX1_Ctrl_M38	Ctrl	+/-	f	M38
B4galnt2_ABX1_Ctrl_M39	Ctrl	+/-	f	M39
B4galnt2_ABX1_Ctrl_M40	Ctrl	+/-	m	M40

Sample_ID	Group	Genotype	Sex	Mouse_ID
B4galnt2_ABX1_Ctrl_M41	Ctrl	+/-	f	M41
B4galnt2_ABX1_Ctrl_M42	Ctrl	+/-	f	M42
B4galnt2_ABX1_STR_M43	STR	-/-	m	M43
B4galnt2_ABX1_STR_M44	STR	-/-	m	M44
B4galnt2_ABX1_STR_M45	STR	-/-	f	M45
B4galnt2_ABX1_STR_M46	STR	-/-	m	M46
B4galnt2_ABX1_STR_M47	STR	-/-	m	M47
B4galnt2_ABX1_STR_M48	STR	-/-	f	M48
B4galnt2_ABX1_STR_M49	STR	-/-	m	M49
B4galnt2_ABX1_STR_M50	STR	+/-	f	M50
B4galnt2_ABX1_STR_M51	STR	+/-	f	M51
B4galnt2_ABX1_STR_M52	STR	+/-	m	M52
B4galnt2_ABX1_STR_M53	STR	+/-	f	M53
B4galnt2_ABX1_STR_M54	STR	+/-	f	M54
B4galnt2_ABX1_STR_M55	STR	+/-	m	M55
B4galnt2_ABX1_STR_M56	STR	+/-	f	M56
B4galnt2_ABX1_VAN_M57	VAN	-/-	f	M57
B4galnt2_ABX1_VAN_M58	VAN	-/-	f	M58
B4galnt2_ABX1_VAN_M59	VAN	-/-	f	M59
B4galnt2_ABX1_VAN_M60	VAN	-/-	m	M60
B4galnt2_ABX1_VAN_M61	VAN	-/-	m	M61
B4galnt2_ABX1_VAN_M62	VAN	-/-	m	M62
B4galnt2_ABX1_VAN_M63	VAN	-/-	f	M63
B4galnt2_ABX1_VAN_M64	VAN	+/-	m	M64
B4galnt2_ABX1_VAN_M65	VAN	+/-	f	M65
B4galnt2_ABX1_VAN_M66	VAN	+/-	f	M66
B4galnt2_ABX1_VAN_M67	VAN	+/-	m	M67
B4galnt2_ABX1_VAN_M68	VAN	+/-	f	M68
B4galnt2_ABX1_VAN_M69	VAN	+/-	f	M69
B4galnt2_ABX1_VAN_M70	VAN	+/-	m	M70
